# Combining visual acuity with refraction reduces overestimation of myopia prevalence in school screenings: an age-stratified analysis

**DOI:** 10.3389/fmed.2026.1776604

**Published:** 2026-03-19

**Authors:** Ningfeng Li, Yu Jiang, Xu Zhang, Wenzhi Huang, Junyan Zhang, Bozheng Zhang, Zongyin Gao, Yunxia Leng

**Affiliations:** 1 Department of Ophthalmology, Guangzhou First People's Hospital, School of Medicine, South China University of Technology, Guangzhou, China; 2 Department of Ophthalmology, Guangzhou First People's Hospital, Guangzhou Medical University, Guangzhou, China; 3 Clinical Epidemiology and Evidence-Based Medicine, Shanxi Bethune Hospital, Shanxi Academy of Medical Sciences, Taiyuan, China; 4 College of Arts and Sciences, Emory University, Atlanta, GA, United States

**Keywords:** myopia, prevalence, SER + UCVA criterion, non-cycloplegic refraction, spherical equivalent refraction, visual acuity, children and adolescents

## Abstract

**Background:**

School myopia screening commonly employs non-cycloplegic spherical equivalent refraction (SER ≤ −0. 50 D) for its practicality. However, this SER-only approach likely overestimates the prevalence of visually significant myopia, as it cannot distinguish true myopia from accommodative pseudomyopia, especially in younger children. We quantified the disparity between this SER-only criterion and a combined criterion integrating uncorrected visual acuity (UCVA), the SER + UCVA criterion, and examined its variation across age groups.

**Methods:**

This serial cross-sectional study (2018–2021) included 20,750 students aged 5–18 years from Southern China. Myopia was defined using two criteria based on non-cycloplegic measurements in at least one eye: (1) SER-only: SER ≤ −0.50 D; and (2) SER + UCVA: SER ≤ −0.50 D plus age-impaired UCVA (>0.20 logMAR at age 5; >0.00 logMAR at ages ≥6) in the same eye. Age-stratified prevalence estimates were compared.

**Results:**

The SER + UCVA criterion yielded consistently lower prevalence estimates than the SER-only criterion (e.g., 48.89% vs. 59.48% in 2021), corresponding to a 15–21% annual relative overestimation. Crucially, this overestimation demonstrated a strong age gradient. It was most severe in young children, with the SER-only myopia prevalence nearly double the SER + UCVA myopia prevalence at ages 5–6 years (relative difference >50%), and progressively narrowed to approximately 10–15% in adolescents (14–18 years). Notably, a significant acceleration in SER + UCVA myopia prevalence was observed in children aged 7–10 years between 2019 and 2020. Furthermore, from age 10 onwards, SER + UCVA myopia prevalence was significantly higher in females than males (all *P* < 0.001).

**Conclusion:**

Sole reliance on non-cycloplegic SER substantially overestimates the burden of visually significant myopia, especially in younger children. Incorporating UCVA provides a more accurate and functionally relevant metric for public health screening. We advocate for adopting age-specific SER + UCVA criteria in school-based screenings to optimize referral efficiency, with the greatest benefit expected for younger populations where specificity gains are maximal.

## Introduction

Myopia has become a prominent global public health concern, with its prevalence particularly high among East Asian populations (1, 2). Its impact extends beyond refractive error, contributing significantly to the overall burden of vision impairment (3). Notably, vision loss from all causes is projected to affect hundreds of millions globally by 2050 (4), with myopia itself forecasted to affect nearly half the world's population, and almost 10% progressing to high myopia (5). This progression is critical because high myopia is associated with sight-threatening pathologies such as retinal detachment, macular hemorrhage, and posterior staphyloma, which can lead to irreversible vision loss and impose substantial socio-economic burdens (6, 7). The rising incidence and earlier onset of myopia in children are especially alarming, as earlier onset is a strong predictor of faster progression and higher final myopic refractive error, thereby elevating the lifetime risk of severe ocular complications (8, 9).

In response, school-based vision screening programs have been widely adopted for early detection. Most large-scale screenings utilize non-cycloplegic spherical equivalent refraction (SER ≤ − 0.50 D) (10, 11) due to practical advantages in efficiency and cost. However, this method has a key limitation: it cannot distinguish true myopia from accommodative pseudomyopia, particularly in younger children (12). Consequently, reliance on SER-only may misclassify children with normal visual function as myopic, potentially overestimating the burden of visually significant myopia and leading to unnecessary referrals and resource strain.

To improve screening specificity, China's National Health Commission issued the “Appropriate Technical Guidelines for the Prevention and Control of Myopia in Children and Adolescents” in 2019, advocating for a composite criterion termed “screening myopia” (13). This approach integrates uncorrected visual acuity (UCVA) impairment with non-cycloplegic SER, aiming to identify children who have both refractive error and functional vision deficit, thus more accurately targeting those in need of clinical intervention (13, 14). While studies have established methods for refractive error surveys in children (15), the quantitative epidemiological difference between these two diagnostic criteria—non-cycloplegic SER-only vs. the non-cycloplegic SER + UCVA approach—and crucially, how this difference varies across key developmental stages from early childhood to adolescence, remains insufficiently characterized.

Accordingly, this study aimed to fill this knowledge gap by: (1) quantifying the disparity in myopia prevalence estimates derived from the SER-only criterion vs. the “SER + UCVA” criterion, and (2) elucidating the precise age-specific patterns of this discrepancy within a large pediatric cohort in Southern China. The insights gained are essential for refining public health screening protocols and optimizing resource allocation in myopia prevention initiatives.

## Participants and methods

### Study population

This was a serial cross-sectional study utilizing data from annual school-based vision screenings conducted from September to November in four consecutive years (2018–2021). The study population comprised students aged 5–18 years (encompassing kindergarten through high school grades) from 19 schools in the Yingde and Qingcheng districts of Qingyuan City, Guangdong Province, Southern China. Each year, an independent sample of students was recruited from the same pool of grades across these schools; the study did not follow a longitudinal cohort.

Participants were excluded based on the following criteria: (1) Presence of severe ocular diseases (e.g., cataract, glaucoma, congenital anomalies) or a history of intraocular surgery or trauma; (2) Current use of orthokeratology lenses (discontinued the night before screening) or contact lenses (not worn on the day of examination); (3) Diagnosis of severe systemic diseases or cognitive impairments that could hinder cooperation; (4) Refusal to participate by either the student or their legal guardian. Additionally, records with missing critical data (UCVA or autorefraction), implausible age entries, or inconsistent information were excluded from the analysis (see Supplementary Figure S1 for flowchart).

This study was approved by the Ethics Committee of Guangzhou First People's Hospital (Approval No. K-2022-101-01). All procedures involving human participants were performed in accordance with the ethical standards of this committee and with the 1964 Helsinki Declaration and its later amendments. Prior to participation, comprehensive information regarding the study's objectives and procedures was provided to both the participants and their legal guardians. Informed verbal consent was obtained from all participants and their guardians to ensure voluntary involvement. Rigorous measures were implemented to maintain the confidentiality and security of all personal data collected.

### Measurements

Comprehensive data collection included basic demographic information such as name, age, sex, grade, identity card number, and medical history, alongside binocular ocular parameters including UCVA, spherical power, cylindrical power, and axial position for each individual. All screenings were conducted by a team of certified optometrists or ophthalmologists who underwent standardized training. All participants underwent a standardized examination protocol during each screening session. The examination protocol was performed in a fixed order under controlled ambient lighting conditions. Visual acuity was assessed using a standardized Chinese national standard logMAR E-chart (GB11533-2011) at a distance of 5 m. Participants were instructed to cover one eye without applying pressure. The smallest line where at least four of five optotypes were correctly identified was recorded as the UCVA for that eye. Non-cycloplegic autorefraction was performed using a desktop autorefractor (Nidek AR-330A, Japan). The participant's chin and forehead were stabilized on the instrument. Three consecutive measurements were taken for each eye. If the spherical equivalent difference between any two measurements exceeded 0.50 diopters (*D*), additional measurements were taken until three readings within this tolerance were obtained. The mean spherical and cylindrical values from these three readings were used for analysis. A brief slit-lamp biomicroscopy and direct ophthalmoscopy were performed to rule out obvious ocular pathology. Students who habitually wore spectacles were required to remove them for both UCVA and autorefraction measurements.

### Definitions

1) Spherical equivalent refraction (SER): Calculated from non-cycloplegic autorefraction as the sphere power plus half of the cylinder power, expressed in diopters (*D*).
2) Definitions of myopia: Myopia was defined according to two distinct criteria applied to at least one eye:
  - SER-only myopia: Non-cycloplegic SER of ≤ -0.50 *D*.
  - SER + UCVA myopia: Non-cycloplegic SER of ≤ -0.50 *D* in an eye accompanied by age-specific UCVA impairment (in the same eye; >0.20 logMAR for age 5; >0.00 logMAR for age 6 and above years), consistent with definitions employed in previous studies (10, 11, 15).
3) Myopia severity classification (based on non-cycloplegic SER at least one eye):
  - Mild myopia: −3.00 *D* < SER ≤ −0.50 *D*.
  - Moderate myopia: −6.00 *D* < SER ≤ −3.00 *D*.
  - High myopia: SER ≤ −6.00 *D*.
4) Visual acuity status classification (based on the worse eye):
  - Normal vision: UCVA of ≤ 0.00 logMAR.
  - Low vision: UCVA > 0.00 logMAR, further stratified into mild (>0.00 and ≤ 0.10 logMAR), moderate (0.20 – 0.40 logMAR), or severe (≥0.50 logMAR) impairment (13).

### Statistical analysis

Continuous variables were represented using the mean and corresponding standard errors or deviations. To compare quantitative data between two calendar years or between variables of the left eye [oculus sinister (OS)] and the right eye [oculus dextrus (OD)], we employed Student's *t*-test and Wilcoxon rank-sum test for normally and abnormally distributed data, respectively. Categorical variables were analyzed using either Chi-square or Fisher's exact test, as appropriate. Prevalence was reported as a proportion along with 95% confidence intervals (95% CIs) to provide a comprehensive understanding of the data. The statistical analyses were performed using Stata SE 13 (Serial number 401306302851), R software version 3.6.1 (http://cran.r-project.org/), and easy-R (www.empowerstats.com). Furthermore, graphical representations were created using GraphPad Prism (http://www.graphpad-prism.cn) to visualize the findings.

## Results

### Subject characteristics

A total of 22,661 initial sup records were collected. After applying exclusion criteria (detailed in Supplementary Figure S1), a final dataset of 20,750 records (41,500 eyes) was included for analysis. The cohort included 5,066 participants in 2018, 5,219 in 2019, 5,356 in 2020, and 5,109 in 2021, aged 5–18 years. The study comprised 11,039 males (53.20%) and 9,711 females (46.80%). Detailed demographic information is provided in Supplementary Table S1 and Supplementary Figure S2.

### Refractive profile and temporal trends

**Figure 1:**
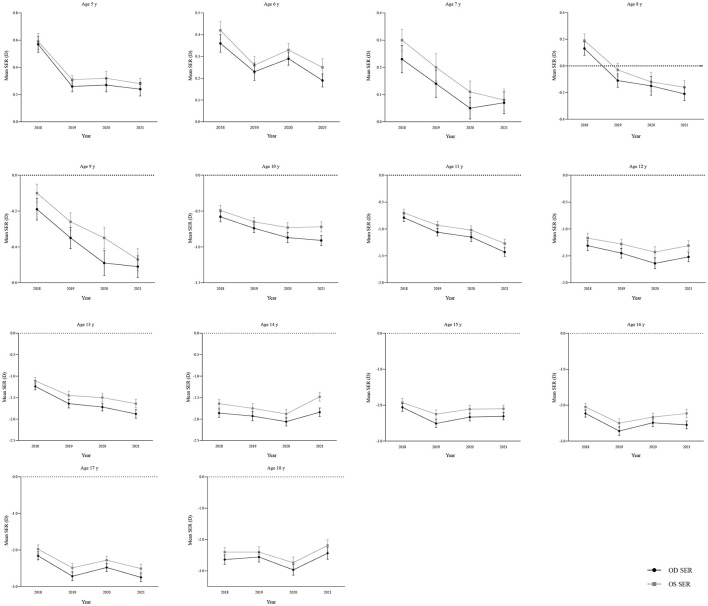
Annual mean spherical equivalent refraction (SER) line graph from 2018 to 2021 stratified by age. SER, spherical equivalent refraction; OD, right eye; OS, left eye. Data points represent the mean non-cycloplegic SER (in diopters, D) for each age cohort. Error bars represent the standard error of the mean. The dotted lines help to show the cross-year trends. The progressive myopic shift, particularly pronounced in younger children (ages 5–11 years), underscores this group's heightened refractive sensitivity during a critical developmental period.

Analysis of the mean non-cycloplegic SER revealed a consistent myopic shift with increasing age and a trend toward more negative SER values over the study period (Table 1 and Figure 1). The right eye generally exhibited a slightly more myopic mean SER than the left eye (Supplementary Table S2). The evolution of the refractive error distribution, visualized via kernel density estimation, substantiated these trends (Supplementary Figure S3). This visualization illustrates the changing refractive landscape: a shrinking emmetropic peak coexists with an expanding myopic tail illustrating the concurrent decline in the emmetropic subpopulation and the growth of the myopic majority. The progressive leftward shift of both the mean SER (vertical line) and the distribution's mass across age and calendar years visually quantifies the population-level myopic shift.

**Table 1:** Annual mean spherical equivalent refraction (SER) of the right eye, stratified by age (2018–2021).

**Age, *y***	**Total**	**SER**								$P_{\text{ab}}^{*}$
		**2018**		**2019**		**2020**		**2021**		
		***N***	**Mean ±SD**	***N***	**Mean ±*SD***	***N***	**Mean ±*SD***	***N***	**Mean ±*SD***	
5	1,012	178	0.57^a^ ± 0.86	311	0.26 ± 0.67	258	0.27 ± 0.77	265	0.24^b^ ± 0.77	**< 0.001**
6	1,701	470	0.36^a^ ± 0.81	396	0.23 ± 0.84	435	0.29 ± 0.61	400	0.19^b^ ± 0.66	**< 0.001**
7	1,676	394	0.23^a^ ± 0.94	439	0.14 ± 1.04	443	0.05^b^ ± 0.79	400	0.07 ± 0.87	**< 0.001**
8	1,710	405	0.13^a^ ± 0.98	437	−0.11 ± 1.07	458	−0.15 ± 1.40	410	−0.21^b^ ± 0.96	**< 0.001**
9	1,715	416	−0.19^a^ ± 1.17	408	−0.35 ± 1.16	443	−0.49 ± 1.38	448	−0.51^b^ ± 1.18	**< 0.001**
10	1,706	432	−0.58^a^ ± 1.45	448	−0.74 ± 1.33	409	−0.87 ± 1.45	417	−0.91^b^ ± 1.48	**< 0.001**
11	1,752	450	−0.79^a^ ± 1.50	450	−1.06 ± 1.56	454	−1.15 ± 1.67	398	−1.43^b^ ± 1.65	**< 0.001**
12	1,581	370	−1.31^a^ ± 1.66	405	−1.45 ± 1.78	392	−1.64^b^ ± 1.98	414	−1.52 ± 1.83	0.052
13	1,557	371	−1.24^a^ ± 1.57	383	−1.64 ± 1.87	420	−1.72 ± 1.84	383	−1.88^b^ ±2.01	**< 0.001**
14	1,467	386	−1.86 ± 1.90	333	−1.93 ± 1.99	360	−2.06^b^ ± 1.92	388	−1.84^a^ ± 2.03	0.061
15	1,425	323	−2.06^a^ ± 2.00	375	−2.51^b^ ± 2.14	376	−2.33 ± 2.10	351	−2.31 ± 1.91	**0.002**
16	1,460	342	−2.23^a^ ± 1.92	351	−2.72^b^ ± 2.20	397	−2.49 ± 2.15	370	−2.55 ± 2.17	**0.003**
17	1,366	345	−2.16^a^ ± 2.11	339	−2.72 ± 2.14	337	−2.48 ± 2.05	345	−2.75^b^ ± 2.25	**0.000**
18	622	184	−2.64 ± 1.99	144	−2.56 ± 1.95	174	−2.97^b^ ± 2.24	120	−2.44^a^ ± 2.06	0.056

SER, spherical equivalent refraction; N, number; SD, standard deviation.

^*a*^stands for maximum SER during 2018–2021; ^*b*^stands for minimum SER during 2018–2021.

^*^Wilcoxon rank-sum test was used to calculate *P*_ab_-value, which represents the statistical difference calculated between the SER maximum and SER minimum for the corresponding year. Bold values represent significance (*P* < 0.05).

### Substantial discrepancy in myopia prevalence between SER-only and SER + UCVA criteria

A direct comparison of the two diagnostic criteria based on non-cycloplegic refraction revealed a significant and consistent discrepancy in estimated myopia burden. The prevalence based on the SER-only criterion was markedly and significantly higher than that based on the SER + UCVA myopia criterion in every study year (Table 2). For instance, in 2021, the SER-only criterion classified 59.48% (95% CI: 58.13–60.82) of children as myopic, whereas the SER + UCVA myopia criterion identified only 48.89% (95% CI: 47.52–50.27). This represents an absolute overestimation of 10.59% points and a relative overestimation of 21.70% by the SER-only criterion. Annually, this discrepancy translated to 384 to 599 fewer children being classified as myopic when functional visual acuity was considered, representing an annual relative overestimation of 15–21% by the SER-only criterion.

**Table 2:** Total prevalence of myopia in 5–18 years of age under different criteria.

**Age, *y***	**Year**	**Total *N***	**SER** + **UCVA myopia**		**SER-only myopia**		**Moderate to high myopia**		**High myopia**	
			***N***	**% (95% CI)**	***N***	**% (95% CI)**	***N***	**% (95% CI)**	***N***	**% (95% CI)**
5	2018	178	5	2.81 (1.16–6.62)	12	6.74 (3.85–11.55)	1	0.56 (0.08–3.95)	0	NA
	2019	311	9	2.89 (1.51–5.49)	42	13.50 (10.12–17.80)	1	0.32 (0.04–2.27)	0	NA
	2020	258	10	3.88 (2.09–7.08)	38	14.73 (10.88–19.63)	0	0.00 (NA–NA)	0	NA
	2021	265	4	1.51 (0.56–3.98)	39	14.72 (10.92–19.55)	1	0.38 (0.05–2.66)	0	NA
6	2018	470	26	5.53 (3.79–8.01)	51	10.85 (8.33–14.01)	3	0.64 (0.21–1.97)	1	0.21 (0.03–1.50)
	2019	396	37	9.34 (6.84–12.65)	66	16.67 (13.30–20.69)	1	0.25 (0.04–1.78)	0	NA
	2020	435	31	7.13 (5.05–9.97)	52	11.95 (9.22–15.37)	1	0.23 (0.03–1.63)	0	NA
	2021	400	48	9.75 (7.20–13.08)	62	15.50 (12.26–19.40)	2	0.50 (0.12–1.99)	0	NA
7	2018	394	37	9.39 (6.87–12.71)	54	13.71 (10.64–17.49)	5	1.27 (0.53–3.02)	2	0.51 (0.13–2.02)
	2019	439	42	8.43 (6.16–11.43)	78	17.77 (14.46–21.64)	4	0.91 (0.34–2.41)	2	0.46 (0.11–1.81)
	2020	443	65	14.67 (11.66–18.30)	106	23.93 (20.17–28.14)	2	0.45 (0.11–1.80)	1	0.23 (0.03–1.60)
	2021	400	51	12.75 (9.81–16.41)	76	19.00 (15.43–23.16)	3	0.75 (0.24–2.31)	1	0.25 (0.03–1.77)
8	2018	405	63	15.56 (12.33–19.44)	86	21.23 (17.51–25.51)	4	0.99 (0.37–2.61)	0	NA
	2019	437	70	16.02 (12.86–19.78)	108	24.71 (20.88–28.99)	12	2.75 (1.56–4.78)	1	0.23 (0.03–1.62)
	2020	458	97	21.18 (17.67–25.18)	133	29.04 (25.05–33.38)	10	2.18 (1.18–4.02)	3	0.66 (0.21–2.02)
	2021	410	99	18.78 (15.27–22.87)	144	35.12 (30.63–39.89)	8	1.95 (0.98–3.86)	1	0.24 (0.03–1.72)
9	2018	416	93	22.36 (18.59–26.63)	126	30.29 (26.05–34.90)	13	3.13 (1.82–5.32)	2	0.48 (0.12–1.91)
	2019	408	101	24.75 (20.79–29.19)	145	35.54 (31.02–40.33)	18	4.41 (2.79–6.91)	2	0.49 (0.12–1.95)
	2020	443	145	32.73 (28.50–37.26)	181	40.86 (36.35–45.52)	25	5.64 (3.84–8.23)	3	0.68 (0.22–2.09)
	2021	448	152	33.93 (29.68–38.46)	197	43.97 (39.42–48.63)	21	4.69 (3.07–7.09)	3	0.67 (0.22–2.06)
10	2018	432	157	36.34 (31.92–41.01)	180	41.67 (37.09–46.40)	37	8.56 (6.26–11.61)	4	0.93 (0.35–2.45)
	2019	448	166	37.05 (32.69–41.64)	220	49.11 (44.48–53.75)	40	8.93 (6.61–11.96)	5	1.12 (0.46–2.66)
	2020	409	198	48.41 (43.58–53.27)	230	56.23 (51.36–60.99)	40	9.78 (7.25–13.08)	2	0.49 (0.12–1.94)
	2021	417	196	47.00 (42.23–51.83)	243	58.27 (53.46–62.94)	44	10.55 (7.94–13.90)	2	0.48 (0.12–1.91)
11	2018	450	197	43.78 (39.24–48.42)	234	52.00 (47.36–56.60)	46	10.22 (7.73–13.39)	3	0.67 (0.21–2.05)
	2019	450	220	48.89 (44.28–53.52)	285	63.33 (58.76–67.68)	57	12.67 (9.89–16.09)	8	1.78 (0.89–3.52)
	2020	454	244	53.74 (49.12–58.30)	293	64.54 (60.01–68.82)	71	15.64 (12.57–19.29)	11	2.42 (1.34–4.33)
	2021	398	249	62.56 (57.68–67.20)	285	71.61 (66.96–75.84)	74	18.59 (15.06–22.74)	6	1.51 (0.68–3.33)
12	2018	370	203	54.86 (49.74–59.89)	247	66.76 (61.77–71.39)	68	18.38 (14.74–22.68)	8	2.16 (1.08–4.28)
	2019	405	213	52.59 (47.70–57.44)	277	68.40 (63.68–72.76)	81	20.00 (16.37–24.20)	13	3.21 (1.87–5.46)
	2020	392	231	58.93 (53.96–63.72)	286	72.96 (68.32–77.14)	101	25.77 (21.66–30.35)	10	2.55 (1.37–4.69)
	2021	414	252	60.87 (56.06–65.48)	312	75.36 (70.96–79.29)	92	22.22 (18.46–26.50)	14	3.38 (2.01–5.64)
13	2018	371	207	55.80 (50.68–60.79)	249	67.12 (62.15–71.73)	57	15.36 (12.03–19.42)	6	1.62 (0.73–3.57)
	2019	383	231	60.31 (55.30–65.12)	283	73.89 (69.24–78.06)	95	24.80 (20.72–29.40)	11	2.87 (1.59–5.12)
	2020	420	272	64.76 (60.05–69.20)	327	77.86 (73.62–81.59)	114	27.14 (23.09–31.62)	12	2.86 (1.63–4.97)
	2021	383	269	70.23 (65.44–74.62)	319	83.29 (79.19–86.71)	104	27.15 (22.92–31.85)	20	5.22 (3.39–7.97)
14	2018	386	260	67.36 (62.50–71.87)	303	78.50 (74.10–82.33)	104	26.94 (22.73–31.61)	17	4.40 (2.75–6.98)
	2019	333	209	62.76 (57.41–67.82)	269	80.78 (76.17–84.68)	91	27.33 (22.79–32.39)	21	6.31 (4.14–9.50)
	2020	360	249	69.17 (64.18–73.74)	298	82.78 (78.50–86.35)	117	32.50 (27.84–37.54)	17	4.72 (2.95–7.48)
	2021	388	259	66.75 (61.89–71.28)	312	80.41 (76.14–84.08)	96	24.74 (20.69–29.30)	20	5.15 (3.34–7.87)
15	2018	323	231	71.52 (66.32–76.20)	261	80.80 (76.12–84.76)	108	33.44 (28.48–38.79)	17	5.26 (3.29–8.32)
	2019	375	284	75.73 (71.11–79.82)	322	85.87 (81.94–89.05)	155	41.33 (36.43–46.41)	30	8.00 (5.64–11.23)
	2020	376	276	73.40 (68.68–77.64)	320	85.11 (81.12–88.37)	134	35.64 (30.94–40.63)	23	6.12 (4.09–9.05)
	2021	351	268	76.35 (71.60–80.53)	310	88.32 (84.50–91.30)	127	36.18 (31.30–41.37)	22	6.27 (4.15–9.35)
16	2018	342	265	77.49 (72.73–81.62)	284	83.04 (78.66–86.67)	126	36.84 (31.87–42.11)	13	3.80 (2.21–6.45)
	2019	351	275	78.35 (73.71–82.37)	312	88.89 (85.13–91.79)	165	47.01 (41.81–52.27)	38	10.83 (7.97–14.55)
	2020	397	307	77.33 (72.93–81.20)	352	88.66 (85.14–91.44)	153	38.54 (33.86–43.44)	35	8.82 (6.39–12.05)
	2021	370	284	76.76 (72.16–80.80)	324	87.57 (83.78–90.57)	151	40.81 (35.89–45.92)	34	9.19 (6.63–12.60)
17	2018	345	257	74.49 (69.60–78.84)	285	82.61 (78.22–86.27)	123	35.65 (30.75–40.88)	19	5.51 (3.53–8.49)
	2019	339	263	77.58 (72.81–81.73)	302	89.09 (85.28–92.00)	150	44.25 (39.02–49.61)	32	9.44 (6.74–13.07)
	2020	337	260	77.15 (72.34–81.34)	301	89.32 (85.52–92.21)	135	40.06 (34.93–45.41)	26	7.72 (5.30–11.11)
	2021	345	270	78.26 (73.57–82.32)	309	89.57 (85.85–92.39)	152	44.06 (38.88–49.37)	35	10.14 (7.36–13.82)
18	2018	184	145	78.80 (72.24–84.16)	161	87.50 (81.83–91.58)	81	44.02 (36.96–51.34)	13	7.07 (4.13–11.84)
	2019	144	117	81.25 (73.94–86.87)	127	88.19 (81.74–92.58)	57	39.58 (31.85–47.88)	9	6.25 (3.26–11.66)
	2020	174	130	74.71 (67.66–80.67)	159	91.38 (86.13–94.76)	77	44.25 (36.98–51.78)	24	13.79 (9.38–19.82)
	2021	120	97	80.83 (72.67–86.99)	107	89.17 (82.11–93.65)	42	35.00 (26.90–44.07)	9	7.50 (3.91–13.91)
Total	2018	5066	2146	42.36 (41.01–43.73)	2533	50.00 (48.62–51.38)	776	15.32 (14.35–16.34)	105	2.07 (1.71–2.50)
	2019	5219	2237	42.86 (41.53–44.21)	2836	54.34 (52.99–55.69)	927	17.76 (16.75–18.82)	172	3.30 (2.84–3.82)
	2020	5356	2515	46.96 (45.62–48.30)	3076	57.43 (56.10–58.75)	980	18.30 (17.28–19.36)	167	3.12 (2.68–3.62)
	2021	5109	2498	48.89 (47.52–50.27)	3039	59.48 (58.13–60.82)	917	17.95 (16.92–19.03)	167	3.27 (2.81–3.79)

SER, spherical equivalent refraction; UCVA, uncorrected visual acuity; N, number; CI, confidence interval.

SER-only myopia: non-cycloplegic SER of ≤ -0.50 *D* at least one eye.

SER + UCVA myopia: non-cycloplegic SER of ≤ -0.50 *D* at least one eye accompanied by age-specific UCVA impairment (in the same eye) (>0.20 logMAR for age 5; >0.00 logMAR for age 6 and above years). Moderate myopia: −6.00 D < SER ≤ −3.00 *D*. High myopia: SER ≤ −6.00 D.

### Pronounced age-dependent pattern of the diagnostic discrepancy

**Figure 2:**
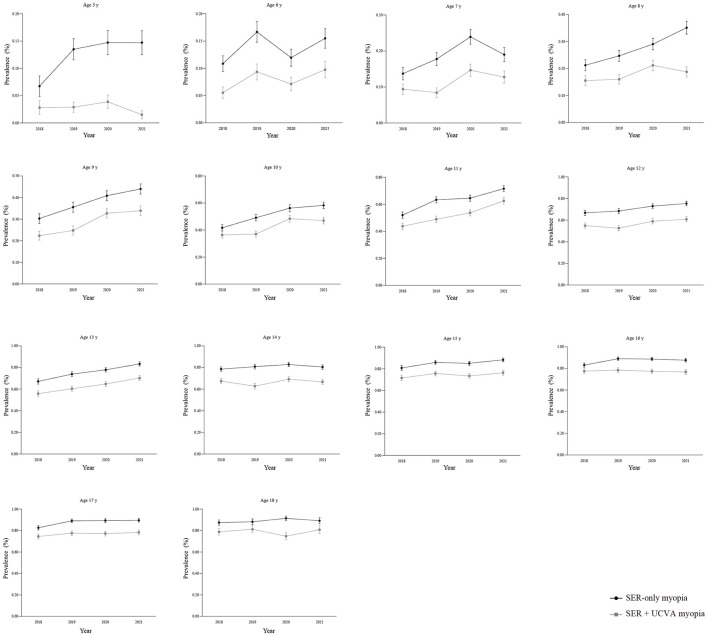
Line plots of the annual prevalence of myopia defined by two criteria from ages 5–18 years. SER, spherical equivalent refraction; UCVA, uncorrected visual acuity. The black line represents the prevalence of SER-only myopia. The gray line represents the prevalence of SER + UCVA myopia. Data points represent the annual prevalence of myopia for each age cohort. Error bars represent the standard error of the mean. SER-only myopia: non-cycloplegic SER of ≤ -0.50 D at least one eye. SER + UCVA myopia: non-cycloplegic SER of ≤ -0.50 D at least one eye accompanied by age-specific UCVA impairment (in the same eye; >0.20 logMAR for age 5; >0.00 logMAR for age 6 and above years). This graph visually demonstrates the differences in prevalence estimation between the two sets of standards, especially in younger children.

The divergence between the two prevalence estimates based on non-cycloplegic refraction was not uniform but exhibited a striking age-dependent gradient (Table 2 and Figure 2). The discrepancy was most extreme in the youngest children. At ages 5–6 years, the prevalence according to the SER-only criterion was approximately double that according to the SER + UCVA myopia criterion (e.g., in 2021: 14.70% vs. 1.50% at age 5; 15.50% vs. 9.80% at age 6), corresponding to a relative difference exceeding 50%. This gap narrowed progressively throughout childhood. The most rapid convergence occurred between ages 7 and 13. By adolescence (ages 14–18 years), the difference diminished substantially, with the SER-only criterion overestimating prevalence by only approximately 10–15% relative to the screening criterion, indicating a closer alignment of the two metrics in older age groups.

### Accelerated increase in SER + UCVA myopia prevalence in young school-aged children during the COVID-19 period

Analysis of temporal trends in SER+UCVA myopia prevalence uncovered a period of marked acceleration. Children aged 7–10 years experienced a sharp increase in SER + UCVA myopia prevalence from 2019 to 2020 (Table 3). The year-to-year increases for ages 7–10 were all statistically significant (*P*-values: 0.020, 0.048, 0.010, and 0.001, respectively). This period coincides with the implementation of COVID-19 pandemic restrictions. No other age group showed a similarly consistent and significant surge over this specific interval.

**Table 3:** Analysis of the difference between calendar years (2018–2021) in the annual prevalence of SER + UCVA myopia.

**Age, *y***	**6**	**7**	**8**	**9**	**10**	**11**	**12**	**13**	**14**	**15**	**16**	**17**	**18**
P^**^ for trend	*t* = 1.32	*t* = 1.49	*t* = 1.52	*t* = 4.77	*t* = 2.57	*t* = 9.29	*t* = 2.15	*t* = 28.10	*t* = 0.32	*t* = 1.42	*t* = −1.15	*t* = 2.29	*t* = −0.03
	*P* = 0.317	*P* = 0.275	*P* = 0.269	***P*** = **0.041**	*P* = 0.124	***P*** = **0.011**	*P* = 0.165	***P*** = **0.001**	*P* = 0.781	*P* = 0.293	*P* = 0.370	*P* = 0.149	*P* = 0.981
P^*^ (2018–2019)	**0.031**	0.931	0.854	0.417	0.827	0.124	0.526	0.209	0.197	0.207	0.784	0.344	0.584
P^*^ (2019–2020)	0.244	**0.020**	**0.048**	**0.010**	**0.001**	0.144	0.072	0.193	0.075	0.464	0.738	0.894	0.163
P^*^ (2020–2021)	**0.016**	0.418	0.297	0.705	0.685	**0.009**	0.574	0.098	0.480	0.360	0.850	0.728	0.219

SER, spherical equivalent refraction; UCVA, uncorrected visual acuity.

SER + UCVA myopia: non-cycloplegic SER of ≤ -0.50 D at least one eye accompanied by age-specific UCVA impairment (in the same eye; >0.20 logMAR for age 5; >0.00 logMAR for age 6 and above years).

^*^P-values were calculated by one-way analysis of variance and Bonferroni correction.

^**^Linear regression was used to calculate *P*-value. Bold values represent significance (*P* < 0.05).

### Sex differences in the prevalence of SER + UCVA myopia

From the age of 10 years onwards, a significant sex disparity in the prevalence of SER + UCVA myopia based on non-cycloplegic refraction emerged and persisted through adolescence (Table 4). Females had a consistently and significantly higher prevalence of SER + UCVA myopia than males across all age groups from 10 to 18 years (all *P* < 0.001). For example, at age 15, the prevalence was 79.30% in females compared to 69.40% in males.

**Table 4:** Prevalence of SER + UCVA myopia stratified by age in the overall 4-years.

**Age, *y***	**Total**	**Males**	**Females**	**χ^2^**	*P* ^*^
5	2.77% (28/1,012)	1.99% (11/553)	3.70% (17/459)	2.7407	0.098
6	8.35% (142/1,701)	8.32% (77/926)	8.39% (65/775)	0.0028	0.958
7	11.63% (195/1,676)	11.37% (103/906)	11.95% (92/770)	0.1359	0.712
8	19.24% (329/1,710)	18.62% (176/945)	20.00% (153/765)	0.5149	0.473
9	28.63% (491/1,715)	27.14% (250/921)	30.35% (241/794)	2.1479	0.143
10	42.03% (717/1,706)	37.08% (343/925)	47.89% (374/781)	20.2958	**< 0.001**
11	51.94% (910/1,752)	45.67% (422/924)	58.94% (488/828)	30.7876	**< 0.001**
12	56.86% (899/1,581)	51.75% (429/829)	62.50% (470/752)	18.5802	**< 0.001**
13	62.80% (979/1,557)	56.39% (463/821)	70.11% (516/736)	31.2702	**< 0.001**
14	66.60% (977/1,467)	62.25% (498/800)	71.81% (479/667)	14.9569	**< 0.001**
15	74.32% (1,059/1,425)	69.44% (500/720)	79.29% (559/705)	18.0929	**< 0.001**
16	77.47% (1,131/1,460)	72.64% (515/709)	82.02% (616/751)	18.407	**< 0.001**
17	76.87% (1,050/1,366)	72.34% (510/705)	81.69% (540/661)	16.7864	**< 0.001**
18	78.62% (489/622)	72.39% (257/355)	86.89% (232/267)	19.0514	**< 0.001**

SER, spherical equivalent refraction; UCVA, uncorrected visual acuity.

SER + UCVA myopia: non-cycloplegic SER of ≤ -0.50 *D* at least one eye accompanied by age-specific UCVA impairment (in the same eye; >0.20 logMAR for age 5; >0.00 logMAR for age 6 and above years).

^*^Pearson's chi-squared was used to calculate *P*-value, which represents the statistical difference between the sexes in the overall 4-year prevalence of screening myopia. Bold values represent significance (*P* < 0.05).

### Distribution of myopia severity and visual acuity status

**Figure 3:**
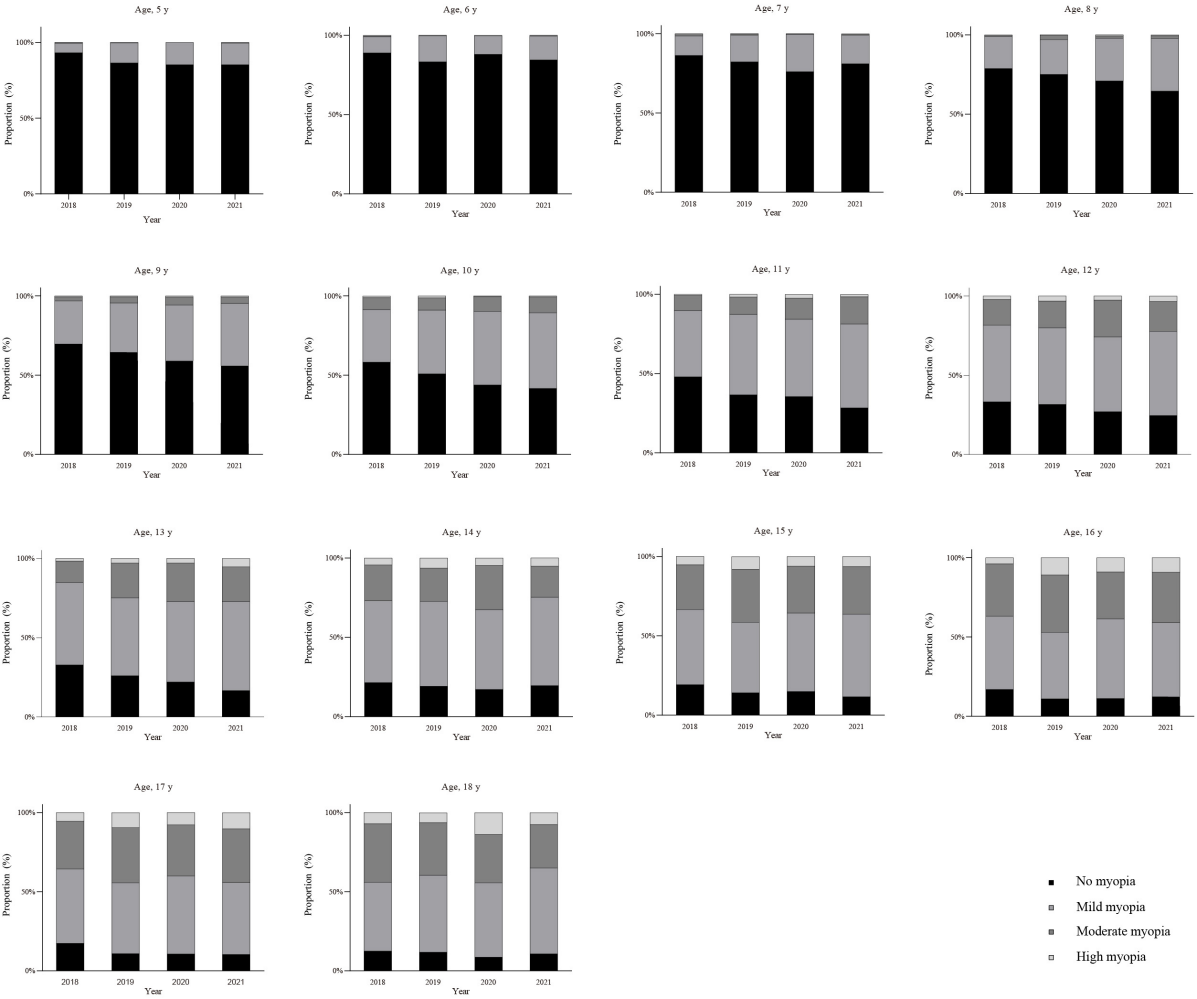
Proportion of different refractive status based on non-cycloplegic spherical equivalent refraction (SER) at different age groups (5–18 years). Severity grades (based on non-cycloplegic SER at least one eye): non-myopia >-0.50 D; mild myopia: −3.00 D < SER ≤ −0.50 D; moderate myopia: −6.00 D < SER ≤ −3.00 D; high myopia: SER ≤ −6.00 D. This figure illustrates the underlying burden of refractive error, which serves as the substrate from which different screening criteria (Figure 2) identify their respective subsets.

The proportion of non-myopic children (SER > −0.50 D) declined steadily with age, falling below 50% after age 10 (Figure 3 and Supplementary Table S3). Concurrently, the proportions of mild, moderate, and high myopia increased. The overall prevalence of high myopia (non-cycloplegic SER ≤ −6.00 D) increased from 2.07% (95% CI: 1.71–2.50) in 2018 to 3.27% (95% CI: 2.81–3.79) in 2021 (Table 2). The prevalence of reduced uncorrected visual acuity (UCVA > 0.00 logMAR) was substantial, exceeding 50% from age 6 and reaching approximately 70% by age 12. The distribution of visual acuity severity grades (mild, moderate, severe) also shifted significantly from 2018 to 2021 for most age groups (Table 5).

**Table 5:** Distribution of visual acuity status stratified by age.

**Age, *y***	**VA. ALERT**	**2018**	**2019**	**2020**	**2021**	*P* ^*^
6	Normal	236 (50.21%)	221 (55.81%)	201 (46.21%)	165 (41.25%)	**< 0.001**
	Low VA					
	Mild	124 (26.38%)	106 (26.77%)	154 (35.40%)	152 (38.00%)	
	Moderate	87 (18.51%)	56 (14.14%)	71 (16.32%)	77 (19.25%)	
	Severe	23 (4.89%)	13 (3.28%)	9 (2.07%)	6 (1.50%)	
7	Normal	215 (54.57%)	298 (67.88%)	256 (57.79%)	207 (51.75%)	**< 0.001**
	Low VA					
	Mild	78 (19.80%)	81 (18.45%)	102 (23.02%)	127 (31.75%)	
	Moderate	70 (17.77%)	45 (10.25%)	68 (15.35%)	61 (15.25%)	
	Severe	31 (7.87%)	15 (3.42%)	17 (3.84%)	5 (1.25%)	
8	Normal	219 (54.07%)	278 (63.62%)	259 (56.55%)	224 (54.63%)	**< 0.001**
	Low VA					
	Mild	61 (15.06%)	76 (17.39%)	103 (22.49%)	107 (26.10%)	
	Moderate	79 (19.51%)	51 (11.67%)	56 (12.23%)	58 (14.15%)	
	Severe	46 (11.36%)	32 (7.32%)	40 (8.73%)	21 (5.12%)	
9	Normal	241 (57.93%)	245 (60.05%)	210 (47.40%)	213 (47.54%)	**0.001**
	Low VA				
	Mild	58 (13.94%)	49 (12.01%)	83 (18.74%)	89 (19.87%)
	Moderate	63 (15.14%)	60 (14.71%)	73 (16.48%)	82 (18.30%)
	Severe	54 (12.98%)	54 (13.24%)	77 (17.38%)	64 (14.29%)
10	Normal	207 (47.92%)	243 (54.24%)	155 (37.90%)	166 (39.81%)	**< 0.001**
	Low VA					
	Mild	54 (12.50%)	50 (11.16%)	49 (11.98%)	62 (14.87%)	
	Moderate	60 (13.89%)	66 (14.73%)	84 (20.54%)	89 (21.34%)	
	Severe	111 (25.69%)	89 (19.87%)	121 (29.58%)	100 (23.98%)	
11	Normal	186 (41.33%)	196 (43.56%)	167 (36.78%)	125 (31.41%)	**0.005**
	Low VA					
	Mild	33 (7.33%)	44 (9.78%)	55 (12.11%)	34 (8.54%)	
	Moderate	83 (18.44%)	71 (15.78%)	83 (18.28%)	91 (22.86%)	
	Severe	148 (32.89%)	139 (30.89%)	149 (32.82%)	148 (37.19%)	
12	Normal	138 (37.30%)	175 (43.21%)	133 (33.93%)	121 (29.23%)	**< 0.001**
	Low VA					
	Mild	21 (5.68%)	25 (6.17%)	37 (9.44%)	52 (12.56%)	
	Moderate	58 (15.68%)	70 (17.28%)	60 (15.31%)	89 (21.50%)	
	Severe	153 (41.35%)	135 (33.33%)	162 (41.33%)	152 (36.71%)	
13	Normal	120 (32.35%)	137 (35.77%)	122 (29.05%)	93 (24.28%)	**< 0.001**
	Low VA					
	Mild	32 (8.63%)	26 (6.79%)	33 (7.86%)	51 (13.32%)	
	Moderate	57 (15.36%)	51 (13.32%)	67 (15.95%)	91 (23.76%)	
	Severe	162 (43.67%)	169 (44.13%)	198 (47.14%)	148 (38.64%)	
14	Normal	96 (24.87%)	110 (33.03%)	97 (26.94%)	112 (28.87%)	**0.049**
	Low VA					
	Mild	29 (7.51%)	25 (7.51%)	34 (9.44%)	29 (7.47%)	
	Moderate	59 (15.28%)	47 (14.11%)	50 (13.89%)	80 (20.62%)	
	Severe	202 (52.33%)	151 (45.35%)	179 (49.72%)	167 (43.04%)	
15	Normal	77 (23.84%)	79 (21.07%)	89 (23.67%)	75 (21.37%)	**0.008**
	Low VA					
	Mild	11 (3.41%)	28 (7.47%)	23 (6.12%)	26 (7.41%)	
	Moderate	40 (12.38%)	40 (10.67%)	48 (12.77%)	69 (19.66%)	
	Severe	195 (60.37%)	228 (60.80%)	216 (57.45%)	181 (51.57%)	
16	Normal	51 (14.91%)	67 (19.09%)	81 (20.40%)	76 (20.54%)	0.081
	Low VA					
	Mild	21 (6.14%)	19 (5.41%)	22 (5.54%)	35 (9.46%)	
	Moderate	45 (13.16%)	38 (10.83%)	50 (12.59%)	53 (14.32%)	
	Severe	225 (65.79%)	227 (64.67%)	244 (61.46%)	206 (55.68%)	
17	Normal	71 (20.58%)	66 (19.47%)	68 (20.18%)	67 (19.42%)	0.285
	Low VA					
	Mild	22 (6.38%)	17 (5.01%)	13 (3.86%)	18 (5.22%)	
	Moderate	29 (8.41%)	36 (10.62%)	43 (12.76%)	53 (15.36%)	
	Severe	223 (64.64%)	220 (64.90%)	213 (63.20%)	207 (60.00%)	
18	Normal	34 (18.48%)	26 (18.06%)	42 (24.14%)	22 (18.33%)	0.050
	Low VA					
	Mild	5 (2.72%)	6 (4.17%)	1 (0.57%)	4 (3.33%)	
	Moderate	20 (10.87%)	24 (16.67%)	15 (8.62%)	24 (20.00%)	
	Severe	125 (67.93%)	88 (61.11%)	116 (66.67%)	70 (58.33%)	

VA, visual acuity.

Visual acuity status classification (based on the worse eye): normal vision: UCVA of ≤ 0.00 logMAR. Low vision: UCVA >0.00 logMAR, further stratified into mild (>0.00 and ≤ 0.10 logMAR), moderate (0.20–0.40 logMAR), or severe (≥0.50 logMAR) impairment.

^*^Pearson's chi-squared was used to calculate *P*-value, which represents the statistical difference in the composition ratio of normal vision, mild low vision, moderate low vision and severe low vision within the same age group over a 4-year period. Bold values represent significance (*P* < 0.05).

## Discussion

### Principal findings

This study provides a critical, large-scale comparison of two prevalent myopia screening definitions. Our data confirm that reliance on non-cycloplegic spherical equivalent refraction alone (SER-only) results in a consistent and substantial overestimation of myopia prevalence—by 15–21% annually—compared to the combined non-cycloplegic “SER + UCVA myopia” criterion, which integrates UCVA. Crucially, we elucidated a pronounced age-dependent pattern in this discrepancy. The overestimation in young children (ages 5–6), where SER-only prevalence was nearly double that of SER + UCVA criterion, strongly suggests the inclusion of a substantial subset with accommodative pseudomyopia or subclinical refractive error (12). The progressive convergence of prevalence estimates with age likely mirrors the transition from dynamic, accommodation-influenced refraction toward stabilized axial myopia, which is more consistently associated with functionally reduced UCVA. Thus, the SER + UCVA criterion acts as a vital specificity filter for immediate referral, better approximating the burden of visually consequential myopia that requires prompt intervention (14). This justifies its role as the primary gatekeeper in the proposed two-tiered screening pathway.

### The rationale and physiological basis for a combined screening criterion

The pathophysiological relationship between SER and UCVA is complex and non-linear. A specific degree of refractive error does not uniformly predict a corresponding level of visual function due to significant inter-individual variability in fundamental optical and physiological factors. These include the depth of focus of the eye and the dynamics of the accommodative system, which determine how optical defocus translates into perceived blur (16). This foundational discordance is empirically well-documented in screening contexts. For instance, a detailed analysis in a Chinese pediatric population demonstrated that a substantial proportion of children with mild myopic SER could retain normal or near-normal UCVA, highlighting the limitations of using refractive error alone as a proxy for functional visual impairment (17). This evidence underscores that SER-only is an imperfect surrogate for the presence of a visually significant condition. Therefore, the SER + UCVA criterion is not merely an additive rule but a pragmatic tool designed to identify children at the clinically meaningful intersection of quantifiable optical defect and measurable functional deficit. By requiring both components, this criterion selectively flags cases where the refractive error is likely to be of immediate functional concern, thereby increasing the clinical relevance and positive predictive value of screening referrals. It intentionally prioritizes specificity to identify children who are most probable to benefit from and require professional intervention based on current visual performance. It is important to acknowledge that the definitive accuracy of this or any non-cycloplegic screening criterion can only be established against cycloplegic refraction. While our study cannot provide such validation metrics, it directly addresses a pragmatic public health question: quantifying the operational difference between two widely used screening definitions in real-world practice.

### Public health and clinical implications

Large-scale school-based screening primarily aims to efficiently identify children with a high probability of visually significant refractive error requiring professional care, thereby optimizing the use of limited clinical resources. Our findings indicate that the SER + UCVA criterion fulfills this purpose by substantially improving screening specificity—particularly in younger children, in whom accommodative pseudomyopia frequently leads to false-positive results. This addresses a major public health challenge: reducing system overload and unnecessary referrals. An inherent trade-off exists, however, between sensitivity and specificity in any screening program. The combined criterion may not flag children with mildly myopic SER but normal UCVA, potentially delaying the identification of early or “pre-myopic” cases. To balance the need for efficient resource allocation with early risk detection, we propose an evidence-based, two-tiered screening and management pathway: (1) Primary Referral: Children meeting the SER + UCVA criterion (SER ≤ −0.50 *D* with age-impaired UCVA) should be referred promptly for comprehensive ophthalmological assessment. (2) Secondary Surveillance: Children meeting only the SER-only criterion (SER ≤ −0.50 *D* with normal UCVA) should be classified as “at-risk” and enrolled in a structured monitoring program. This includes targeted health education on myopia prevention (e.g., outdoor activity, visual hygiene) and prioritized re-screening within 6–12 months to detect progression to functionally significant myopia. This stratified approach allows the SER + UCVA criterion to serve as the cornerstone of a cost-effective, multi-step screening system—enhancing specificity for immediate referrals while systematically tracking at-risk children for early preventive intervention.

### Age-related susceptibility and environmental drivers of refractive trends

Our analysis reveals two interconnected epidemiological patterns: a progressive, population-level myopic shift across all ages from 2018 to 2021, most pronounced in younger children (5–11 years, Figure 1), and a discrete surge in the prevalence of functionally significant (SER + UCVA) myopia specifically among children aged 7–10 years between 2019 and 2020.

Younger school-aged children are in a critical period of ocular development characterized by heightened plasticity of the emmetropization process and rapid axial elongation (18). This renders their refractive state particularly vulnerable to myopiagenic environmental stimuli. The sustained, progressive trend observed from 2018 onward is primarily attributable to chronic, endemic risk factors in the population. These include pervasive and sustained pressures such as intensive educational demands, prolonged near work, and limited outdoor time—well-established drivers of the secular rise in myopia prevalence in societies (19–21).

Superimposed on this chronic baseline was an acute, intensive environmental perturbation: the COVID-19 pandemic lockdowns initiated in early 2020. This event constituted a dramatic, albeit likely temporary, amplification of key risk factors (notably near-work and indoor confinement) while eliminating protective outdoor activity. For children within this sensitive developmental window, this acted as a potent exogenous stressor, directly explaining the marked 2019–2020 surge observed in this cohort, consistent with reports of a short-term “lockdown spike” (22, 23).

However, the continuous progression of the mean SER through 2021 indicates that the pandemic's impact cannot explain the entire trend. Longitudinal evidence suggests such acute spikes may be partially reversible upon resumption of normal activities, highlighting the transient nature of the behavioral shock (23). Therefore, the overarching 2018–2021 trend is best interpreted as the compound effect of a chronically pro-myopic environment on a susceptible population, upon which an acute exogenous shock was temporarily superimposed. Our cross-sectional design captures the net refractive status of the population under the combined influence of both enduring and temporary factors at each time point and cannot isolate individual-level recovery, which may explain the apparent divergence from a pure “transient spike” hypothesis.

Definitive attribution is limited by the lack of granular data on local confinement policies in Qingyuan and the inherent constraints of the serial cross-sectional design for delineating transient individual changes. Nevertheless, this integrative model—linking fundamental age-related susceptibility to stratified environmental drivers—provides a coherent explanation for the observed data and underscores the critical importance of addressing sustained environmental risk factors, especially for the most vulnerable younger children, within public health strategies for myopia control.

## Sex disparity

Moreover, the consistent finding that females exhibit a higher prevalence of SER + UCVA myopia from the age of 10 onwards is corroborated by previous epidemiological research (17, 24, 25). Although the precise etiological mechanisms remain to be fully elucidated, this phenomenon may result from a confluence of factors, including earlier biological maturation in females (26–28) and potential sex-related behavioral differences (29–31). These results highlight the importance of tailoring screening programs to account for age- and sex-specific risk profiles.

## Limitations and future directions

This study has several limitations. First, the use of non-cycloplegic autorefraction, while pragmatic for large-scale screening, precludes definitive differentiation between true myopia and accommodative pseudomyopia. Consequently, we cannot ascertain the exact proportion of children identified by the SER-only criterion who had pseudomyopia, nor can we calculate the precise diagnostic sensitivity and specificity of the SER + UCVA criterion against a cycloplegic gold standard. Second, the reliability of measurements, particularly in the youngest children (aged 5–6 years), may be affected by variable cooperation during UCVA testing and autorefraction, potentially influencing the precision of prevalence estimates in this age group. Third, for participants with a history of orthokeratology or contact lens wear, an excessively short washout period may not fully eliminate its effect on corneal curvature, potentially introducing measurement bias. Fourth, the use of the specific autorefractor (Nidek AR-330A) may contribute to instrument accommodation, a known source of myopic shift in non-cycloplegic measurements. Finally, as our sample was drawn from a specific region in Southern China, the generalizability of the observed age-specific patterns to other populations with different genetic and environmental backgrounds warrants further investigation.

Future research should involve validation studies where children identified by different screening algorithms subsequently undergo cycloplegic refraction to establish the optimal test characteristics and refine the SER and UCVA cut-off values for the combined criterion. Longitudinal studies are also needed to evaluate the long-term outcomes of children stratified by the proposed two-tiered screening pathway.

## Conclusion

In conclusion, reliance on non-cycloplegic SER-only leads to a substantial, age-dependent overestimation of visually significant myopia. The composite non-cycloplegic “SER + UCVA myopia” criterion, incorporating functional visual acuity, provides a more clinically relevant and public health-meaningful metric. We advocate for its adoption in school-based screening programs, with enhanced emphasis on its use in younger children to improve screening specificity. Implementing such evidence-based, refined criteria is a crucial step toward optimizing resource allocation, accurately monitoring epidemiological trends, and ultimately prioritizing interventions for children most at risk of vision impairment from myopia.

## Data Availability

The raw data supporting the conclusions of this article will be made available by the authors, without undue reservation.
