# Supplementary material for: Combining visual acuity with refraction reduces overestimation of myopia prevalence in school screenings: an age-stratified analysis
Source: Front Med (Lausanne). 2026 Mar 19;13:1776604. doi: 10.3389/fmed.2026.1776604 (PMC13043352; doi:10.3389/fmed.2026.1776604)
Supplement: Supplementary file 1 [file Data_Sheet_1.docx]

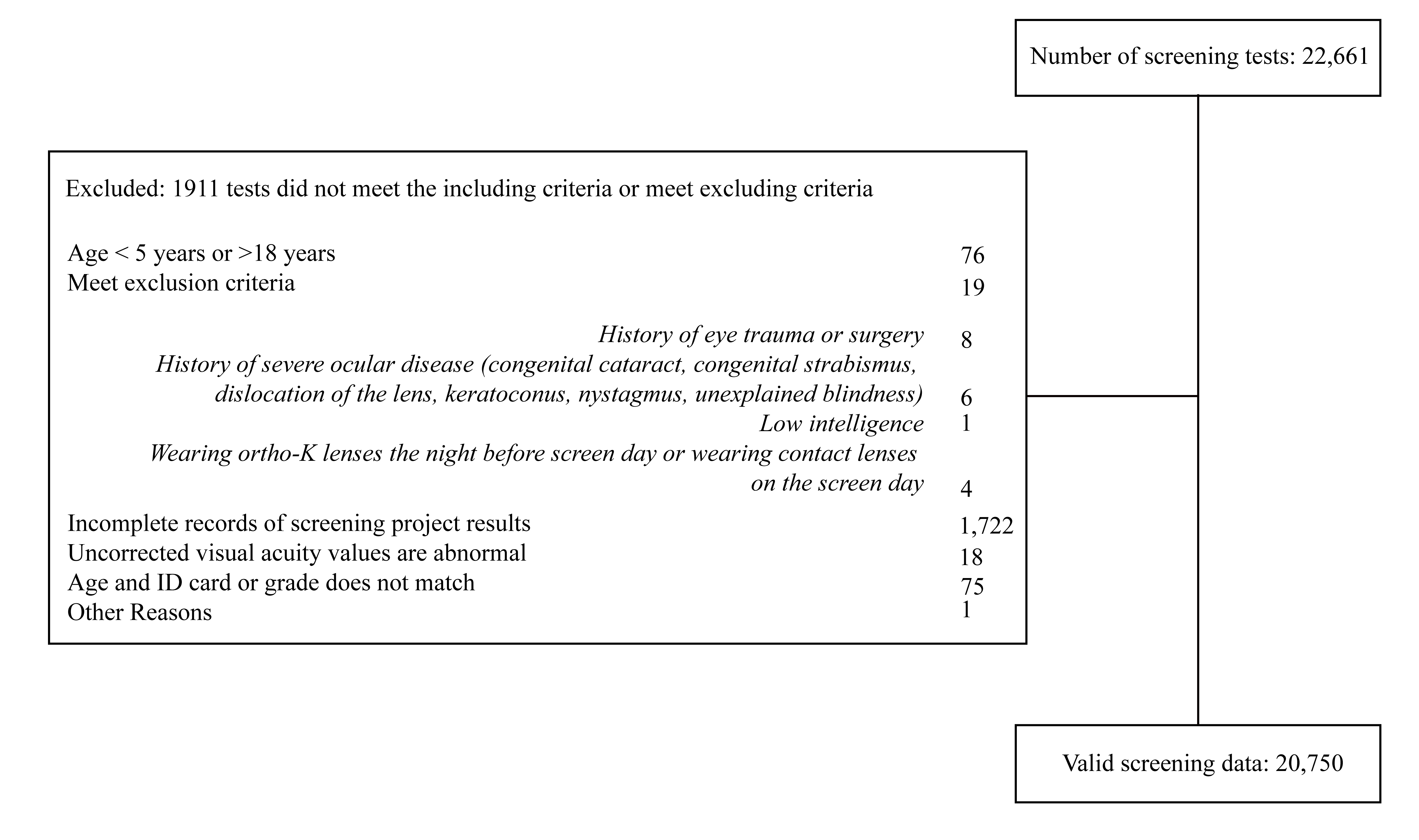


**FIGURE S1. Flow chart for data screening process.**

Schematic diagram detailing the sequential process of screening record review, application of exclusion criteria, and derivation of the final analytic sample.

**TABLE S1. Analysis of sample size, age, and sex distribution by year.**

| Sex, n(%) |  |  |  |  |  |  |
| --- | --- | --- | --- | --- | --- | --- |
|  | Male | 2753 (54.34%) | 2770 (53.08%) | 2760 (51.53%) | 2756 (53.94%) | χ 2=9.82 |
|  | Female | 2313 (45.66%) | 2449 (46.92%) | 2596 (48.47%) | 2353 (46.06%) | * p=**0.020** |
| Age, n(%) |  |  |  |  |  |  |
|  | 5 | 178 (3.51%) | 311 (5.96%) | 258 (4.82%) | 265 (5.19%) |  |
|  | 6 | 470 (9.28%) | 396 (7.59%) | 435 (8.12%) | 400 (7.83%) |  |
|  | 7 | 394 (7.78%) | 439 (8.41%) | 443 (8.27%) | 400 (7.83%) |  |
|  | 8 | 405 (7.99%) | 437 (8.37%) | 458 (8.55%) | 410 (8.03%) |  |
|  | 9 | 416 (8.21%) | 408 (7.82%) | 443 (8.27%) | 448 (8.77%) |  |
|  | 10 | 432 (8.53%) | 448 (8.58%) | 409 (7.64%) | 417 (8.16%) |  |
|  | 11 | 450 (8.88%) | 450 (8.62%) | 454 (8.48%) | 398 (7.79%) |  |
|  | 12 | 370 (7.30%) | 405 (7.76%) | 392 (7.32%) | 414 (8.10%) |  |
|  | 13 | 371 (7.32%) | 383 (7.34%) | 420 (7.84%) | 383 (7.50%) |  |
|  | 14 | 386 (7.62%) | 333 (6.38%) | 360 (6.72%) | 388 (7.59%) |  |
|  | 15 | 323 (6.38%) | 375 (7.19%) | 376 (7.02%) | 351 (6.87%) |  |
|  | 16 | 342 (6.75%) | 351 (6.73%) | 397 (7.41%) | 370 (7.24%) |  |
|  | 17 | 345 (6.81%) | 339 (6.50%) | 337 (6.29%) | 345 (6.75%) |  |
|  | 18 | 184 (3.63%) | 144 (2.76%) | 174 (3.25%) | 120 (2.35%) |  |

* Pearson’s chi-squared was used to calculate P value, which represents the statistical difference between the sexes.

# **
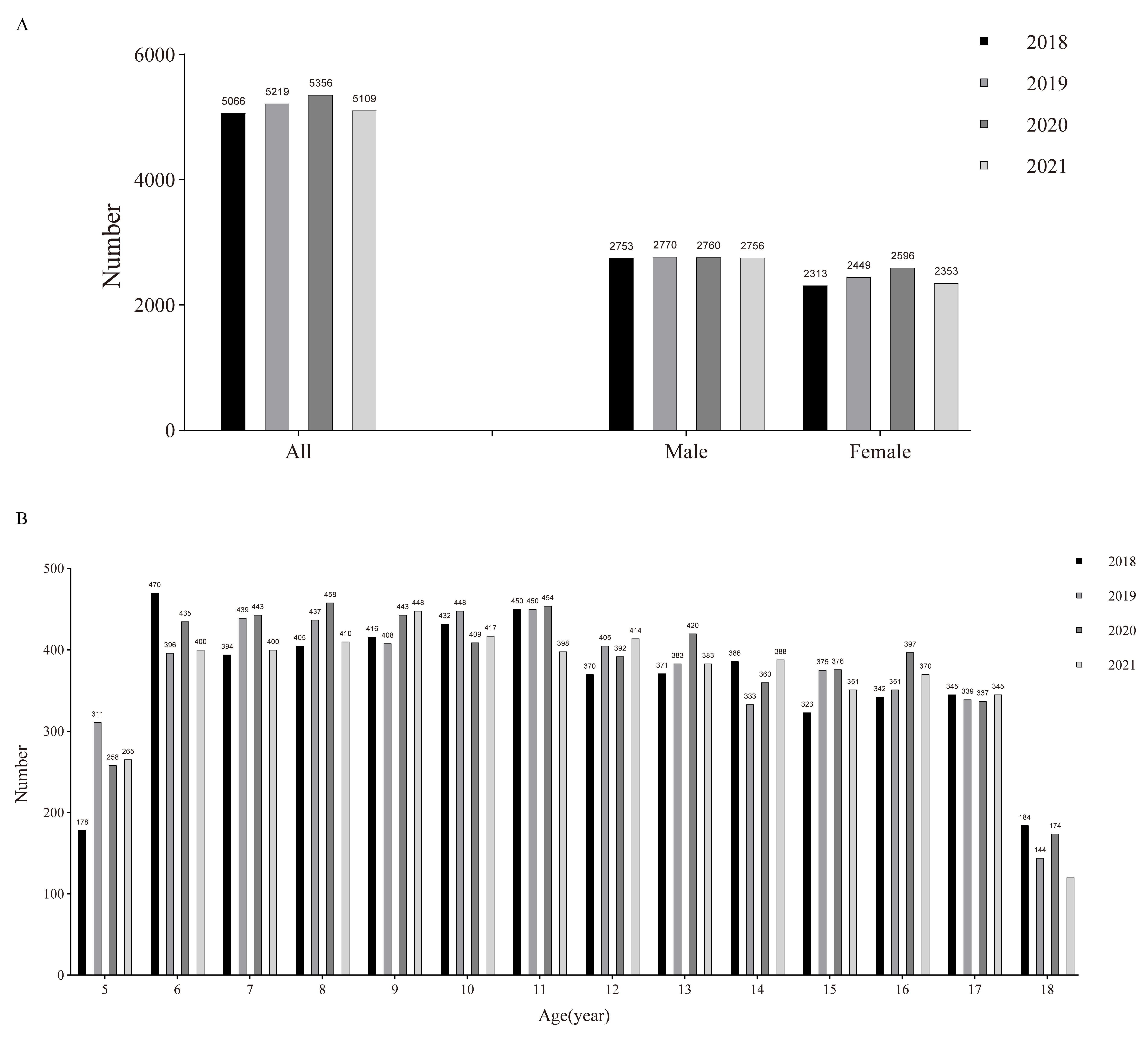
**

# **FIGURE S2. Demographic distribution of the study population by year.**

Histograms showing the sample size composition by age and sex for each cross-sectional study year (2018-2021).

## **TABLE S2. Non-cycloplegic spherical equivalent refraction (SER) of the right and left eyes, stratified by age and year (2018–2021).**

| Age,y | Eye category |  | | | |  | | | | SER | | | |  | | | |  | | | | P-value * | P **  (2018vs2021) | P ***  (2018 vs 2019) | P ***  (2019vs 2020) | P ***  (2020 vs 2021) |
| --- | --- | --- | --- | --- | --- | --- | --- | --- | --- | --- | --- | --- | --- | --- | --- | --- | --- | --- | --- | --- | --- | --- | --- | --- | --- | --- |
|  |  | 2018 | | | | 2019 | | | | 2020 | | | | 2021 | | | | Total (2018-2021) | | | |  |  |  |  |  |
|  |  | N | Mean | SE | SD | N | Mean | SE | SD | N | Mean | SE | SD | N | Mean | SE | SD | N | Mean | SE | SD |  |  |  |  |  |
| 5 | OD | 178 | 0.57^a^ | 0.06 | 0.86 | 311 | 0.26 | 0.04 | 0.67 | 258 | 0.27 | 0.05 | 0.77 | 265 | 0.24 ^b^ | 0.05 | 0.77 | 1012 | 0.31 | 0.02 | 0.77 | **<0.001** | 0.203 | **<0.001** | 0.511 | 0.177 |
|  | OS | 178 | 0.59 ^a^ | 0.06 | 0.87 | 311 | 0.31 | 0.03 | 0.55 | 258 | 0.32 | 0.05 | 0.76 | 265 | 0.28 ^b^ | 0.04 | 0.73 | 1012 | 0.36 | 0.02 | 0.72 | **<0.001** |  | **<0.001** | 0.841 | 0.236 |
| 6 | OD | 470 | 0.36 ^a^ | 0.04 | 0.81 | 396 | 0.23 | 0.04 | 0.84 | 435 | 0.29 | 0.03 | 0.61 | 400 | 0.19 ^b^ | 0.03 | 0.66 | 1701 | 0.27 | 0.02 | 0.74 | **<0.001** | **0.035** | **<0.001** | 0.064 | **0.046** |
|  | OS | 470 | 0.42 ^a^ | 0.04 | 0.8 | 396 | 0.26 | 0.04 | 0.72 | 435 | 0.33 | 0.03 | 0.60 | 400 | 0.25 ^b^ | 0.04 | 0.73 | 1701 | 0.32 | 0.02 | 0.72 | **<0.001** |  | **<0.001** | 0.061 | **0.001** |
| 7 | OD | 394 | 0.23 ^a^ | 0.05 | 0.94 | 439 | 0.14 | 0.05 | 1.04 | 443 | 0.05 ^b^ | 0.04 | 0.79 | 400 | 0.07 | 0.04 | 0.87 | 1676 | 0.12 | 0.02 | 0.92 | **<0.001** | 0.111 | **0.006** | 0.060 | 0.494 |
|  | OS | 394 | 0.30 ^a^ | 0.04 | 0.89 | 439 | 0.20 | 0.05 | 0.97 | 443 | 0.11 | 0.04 | 0.78 | 400 | 0.08 ^b^ | 0.04 | 0.89 | 1676 | 0.17 | 0.02 | 0.89 | **<0.001** |  | **0.001** | 0.125 | 0.215 |
| 8 | OD | 405 | 0.13 ^a^ | 0.05 | 0.98 | 437 | -0.11 | 0.05 | 1.07 | 458 | -0.15 | 0.07 | 1.40 | 410 | -0.21 ^b^ | 0.05 | 0.96 | 1710 | -0.09 | 0.03 | 1.13 | **<0.001** | 0.135 | **0.000** | 0.928 | 0.097 |
|  | OS | 405 | 0.19 ^a^ | 0.05 | 0.96 | 437 | -0.03 | 0.05 | 1.08 | 458 | -0.12 | 0.07 | 1.49 | 410 | -0.16 ^b^ | 0.05 | 0.94 | 1710 | -0.03 | 0.03 | 1.15 | **<0.001** |  | **0.000** | 0.683 | **0.033** |
| 9 | OD | 416 | -0.19 ^a^ | 0.06 | 1.17 | 408 | -0.35 | 0.06 | 1.16 | 443 | -0.49 | 0.07 | 1.38 | 448 | -0.51 ^b^ | 0.06 | 1.18 | 1715 | -0.39 | 0.03 | 1.23 | **<0.001** | **0.030** | **0.025** | 0.221 | 0.339 |
|  | OS | 416 | -0.10 ^a^ | 0.05 | 1.06 | 408 | -0.26 | 0.05 | 1.09 | 443 | -0.35 | 0.06 | 1.22 | 448 | -0.47 ^b^ | 0.06 | 1.21 | 1715 | -0.30 | 0.03 | 1.16 | **<0.001** |  | **0.006** | 0.649 | **0.033** |
| 10 | OD | 432 | -0.58 ^a^ | 0.07 | 1.45 | 448 | -0.74 | 0.06 | 1.33 | 409 | -0.87 | 0.07 | 1.45 | 417 | -0.91 ^b^ | 0.07 | 1.48 | 1706 | -0.77 | 0.03 | 1.43 | **<0.001** | **0.009** | **0.004** | **0.043** | 0.569 |
|  | OS | 432 | -0.49 ^a^ | 0.07 | 1.42 | 448 | -0.65 | 0.06 | 1.30 | 409 | -0.73 ^b^ | 0.07 | 1.38 | 417 | -0.72 | 0.07 | 1.39 | 1706 | -0.65 | 0.03 | 1.38 | **0.000** |  | **0.002** | 0.274 | 0.931 |
| 11 | OD | 450 | -0.79 ^a^ | 0.07 | 1.50 | 450 | -1.06 | 0.07 | 1.56 | 454 | -1.15 | 0.08 | 1.67 | 398 | -1.43 ^b^ | 0.08 | 1.65 | 1752 | -1.10 | 0.04 | 1.61 | **<0.001** | **0.020** | **0.000** | 0.953 | **0.001** |
|  | OS | 450 | -0.70 ^a^ | 0.07 | 1.50 | 450 | -0.93 | 0.07 | 1.54 | 454 | -1.02 | 0.08 | 1.60 | 398 | -1.27 ^b^ | 0.08 | 1.65 | 1752 | -0.97 | 0.04 | 1.58 | **<0.001** |  | **0.003** | 0.651 | **0.006** |
| 12 | OD | 370 | -1.31 ^a^ | 0.09 | 1.66 | 405 | -1.45 | 0.09 | 1.78 | 392 | -1.64 ^b^ | 0.10 | 1.98 | 414 | -1.52 | 0.09 | 1.83 | 1581 | -1.48 | 0.05 | 1.82 | 0.052 | **0.005** | 0.339 | 0.294 | 0.717 |
|  | OS | 370 | -1.17 ^a^ | 0.09 | 1.70 | 405 | -1.28 | 0.09 | 1.83 | 392 | -1.43 ^b^ | 0.10 | 1.92 | 414 | -1.31 | 0.09 | 1.87 | 1581 | -1.30 | 0.05 | 1.84 | 0.084 |  | 0.442 | 0.311 | 0.894 |
| 13 | OD | 371 | -1.24 ^a^ | 0.08 | 1.57 | 383 | -1.64 | 0.10 | 1.87 | 420 | -1.72 | 0.09 | 1.84 | 383 | -1.88 ^b^ | 0.10 | 2.01 | 1557 | -1.63 | 0.05 | 1.84 | **<0.001** | **0.003** | **0.001** | 0.560 | 0.397 |
|  | OS | 371 | -1.11 ^a^ | 0.08 | 1.59 | 383 | -1.45 | 0.10 | 1.88 | 420 | -1.50 | 0.09 | 1.82 | 383 | -1.64 ^b^ | 0.10 | 2.00 | 1557 | -1.43 | 0.05 | 1.84 | **0.000** |  | **0.025** | 0.384 | 0.788 |
| 14 | OD | 386 | -1.86 | 0.10 | 1.90 | 333 | -1.93 | 0.11 | 1.99 | 360 | -2.06 ^b^ | 0.10 | 1.92 | 388 | -1.84 ^a^ | 0.10 | 2.03 | 1467 | -1.92 | 0.05 | 1.96 | 0.061 | **0.001** | 0.953 | 0.135 | 0.061 |
|  | OS | 386 | -1.64 | 0.10 | 1.95 | 333 | -1.75 | 0.11 | 2.09 | 360 | -1.88 ^b^ | 0.11 | 2.08 | 388 | -1.48 ^a^ | 0.10 | 1.94 | 1467 | -1.68 | 0.05 | 2.02 | **0.006** |  | 0.487 | 0.333 | **0.006** |
| 15 | OD | 323 | -2.06 ^a^ | 0.11 | 2.00 | 375 | -2.51 ^b^ | 0.11 | 2.14 | 376 | -2.33 | 0.11 | 2.10 | 351 | -2.31 | 0.10 | 1.91 | 1425 | -2.31 | 0.05 | 2.05 | **0.002** | **0.007** | **0.002** | 0.166 | 0.885 |
|  | OS | 323 | -1.93 ^a^ | 0.12 | 2.17 | 375 | -2.25 ^b^ | 0.12 | 2.24 | 376 | -2.11 | 0.11 | 2.13 | 351 | -2.10 | 0.10 | 1.95 | 1425 | -2.10 | 0.06 | 2.13 | 0.085 |  | 0.085 | 0.333 | 0.698 |
| 16 | OD | 342 | -2.23 ^a^ | 0.10 | 1.92 | 351 | -2.72 ^b^ | 0.12 | 2.20 | 397 | -2.49 | 0.11 | 2.15 | 370 | -2.55 | 0.11 | 2.17 | 1460 | -2.50 | 0.06 | 2.12 | **0.003** | **0.005** | **0.003** | 0.125 | 0.943 |
|  | OS | 342 | -2.05 ^a^ | 0.10 | 1.88 | 351 | -2.50 ^b^ | 0.12 | 2.17 | 397 | -2.33 | 0.11 | 2.15 | 370 | -2.23 | 0.12 | 2.25 | 1460 | -2.28 | 0.06 | 2.13 | **0.010** |  | **0.010** | 0.237 | 0.334 |
| 17 | OD | 345 | -2.16 ^a^ | 0.11 | 2.11 | 339 | -2.72 | 0.12 | 2.14 | 337 | -2.48 | 0.11 | 2.05 | 345 | -2.75 ^b^ | 0.12 | 2.25 | 1366 | -2.53 | 0.06 | 2.15 | **0.000** | **0.013** | **0.000** | 0.173 | 0.101 |
|  | OS | 345 | -1.98 ^a^ | 0.12 | 2.22 | 339 | -2.49 | 0.12 | 2.22 | 337 | -2.28 | 0.11 | 2.09 | 345 | -2.51 ^b^ | 0.12 | 2.29 | 1366 | -2.32 | 0.06 | 2.21 | **0.002** |  | **0.003** | 0.248 | 0.205 |
| 18 | OD | 184 | -2.64 | 0.15 | 1.99 | 144 | -2.56 | 0.16 | 1.95 | 174 | -2.97 ^b^ | 0.17 | 2.24 | 120 | -2.44 ^a^ | 0.19 | 2.06 | 622 | -2.68 | 0.08 | 2.07 | 0.056 | 0.061 | 0.715 | 0.177 | 0.056 |
|  | OS | 184 | -2.40­­ | 0.15 | 2.03 | 144 | -2.40 | 0.17 | 2.03 | 174 | -2.74 ^b^ | 0.18 | 2.31 | 120 | -2.19 ^a^ | 0.18 | 2.02 | 622 | -2.45 | 0.08 | 2.11 | 0.062 |  | 0.949 | 0.287 | 0.062 |

## Abbreviations: SER, spherical equivalent refraction; SE, standard error; N, number; SD, standard deviation.

## ^a^, stands for maximum SER during 2018-2021; ^b^, stands for minimum SER during 2018-2021; Bold values represent significance (P<0.05).

* P-values represent the statistical difference calculated between the SER maximum and SER minimum for the corresponding year.

** P-values indicate the difference between the listed years.

*** P -values referred to the difference between consecutive years.


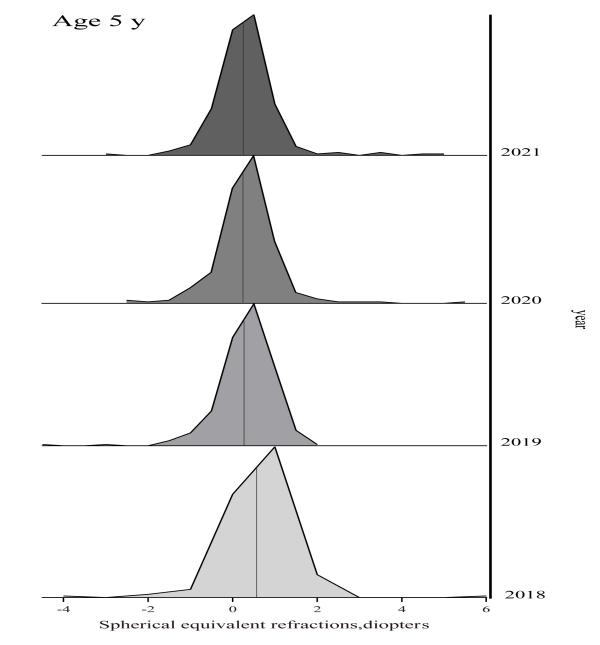

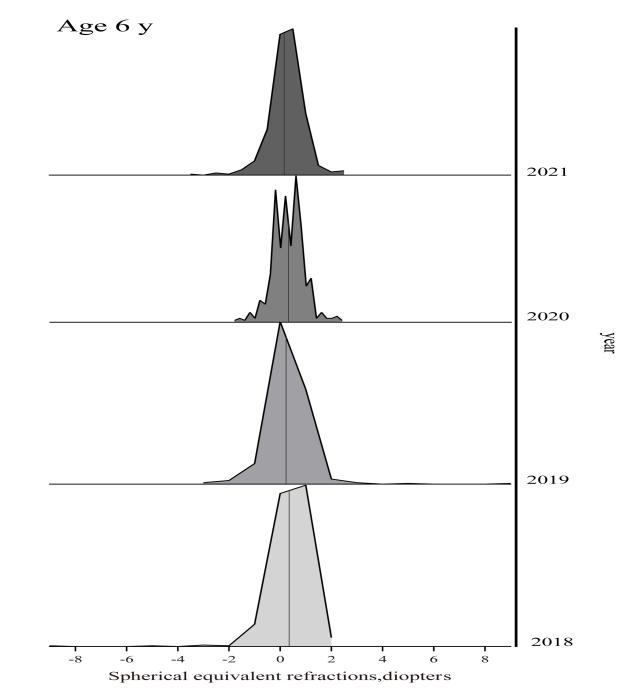

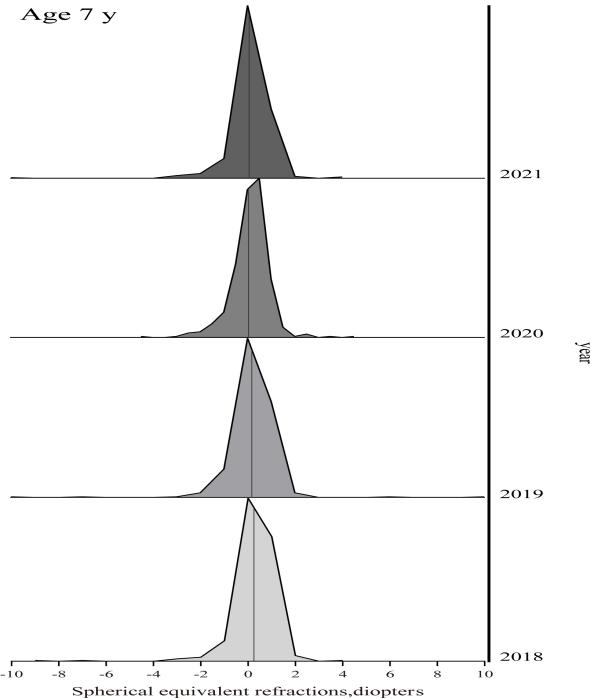

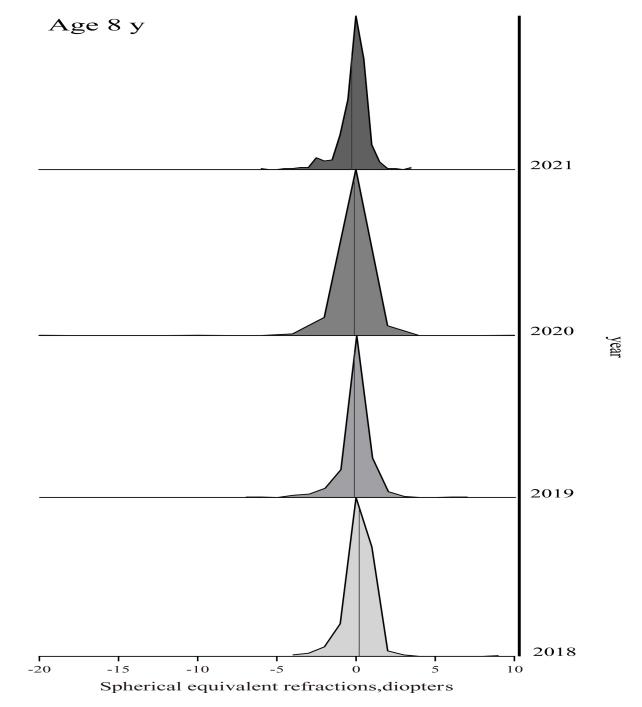

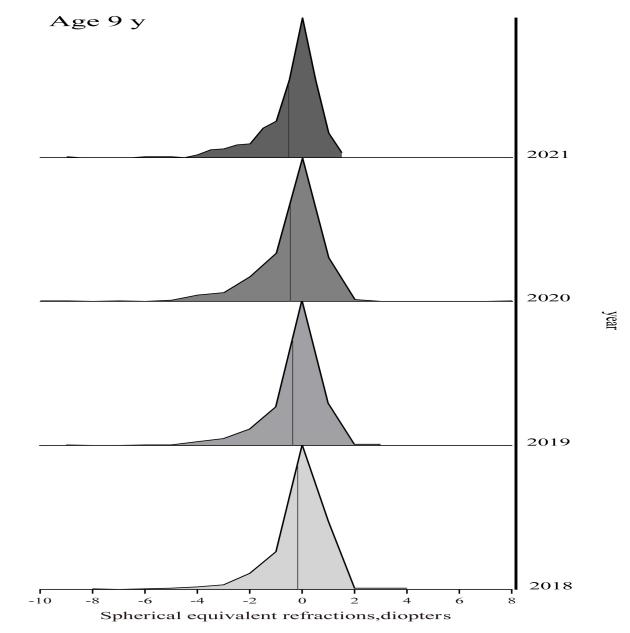

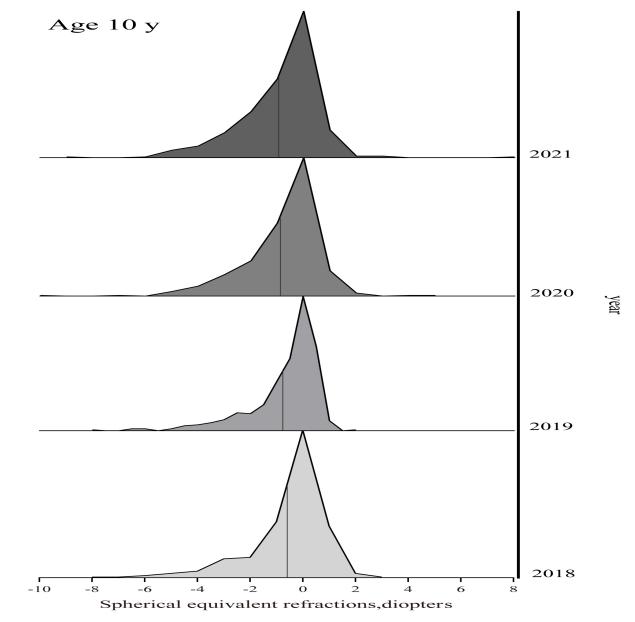

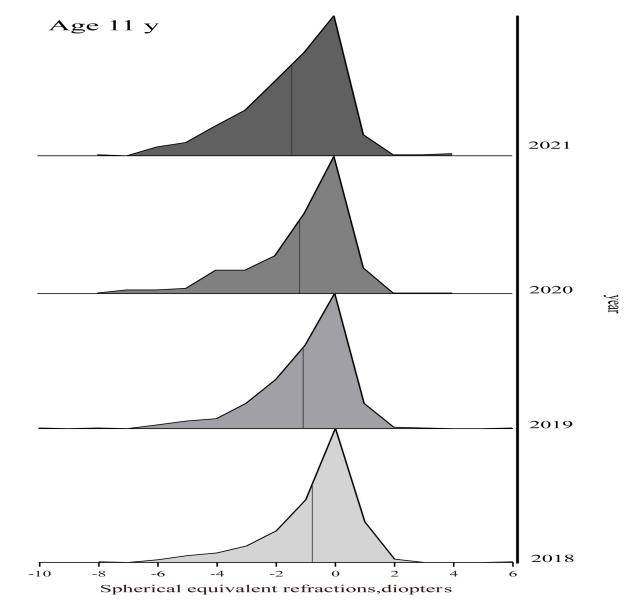

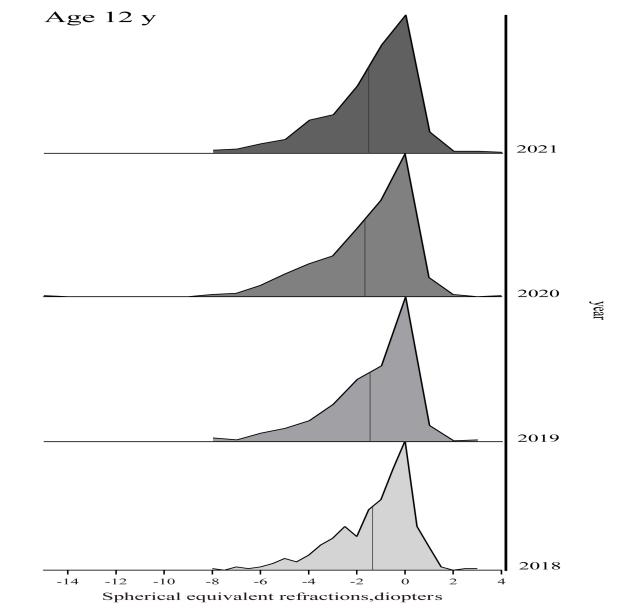

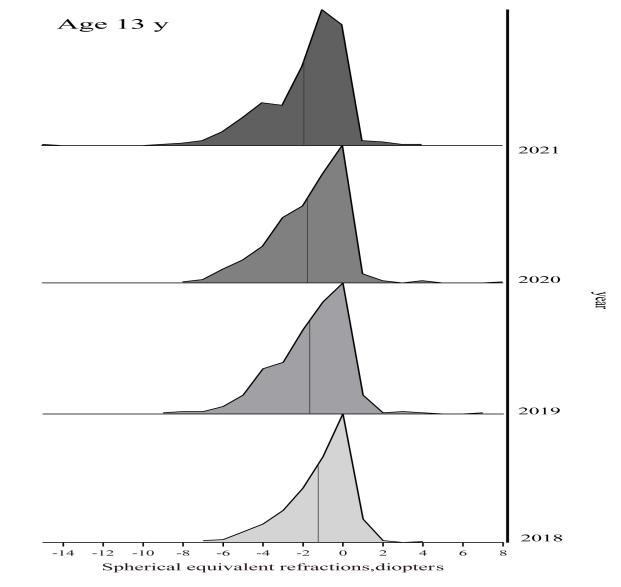

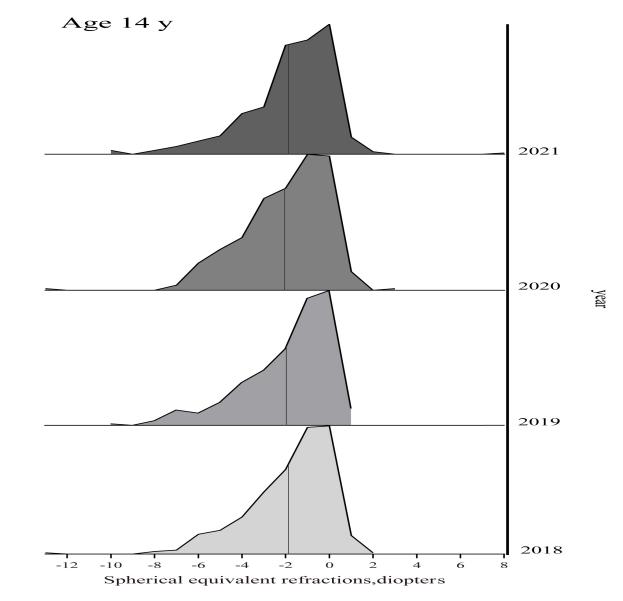

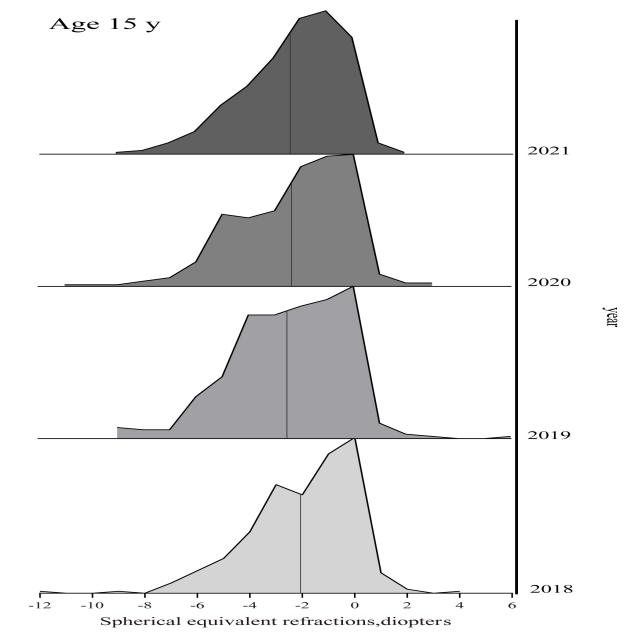

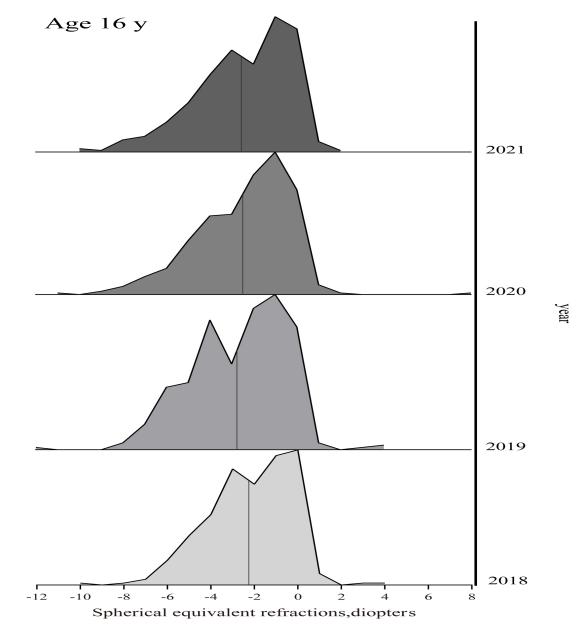

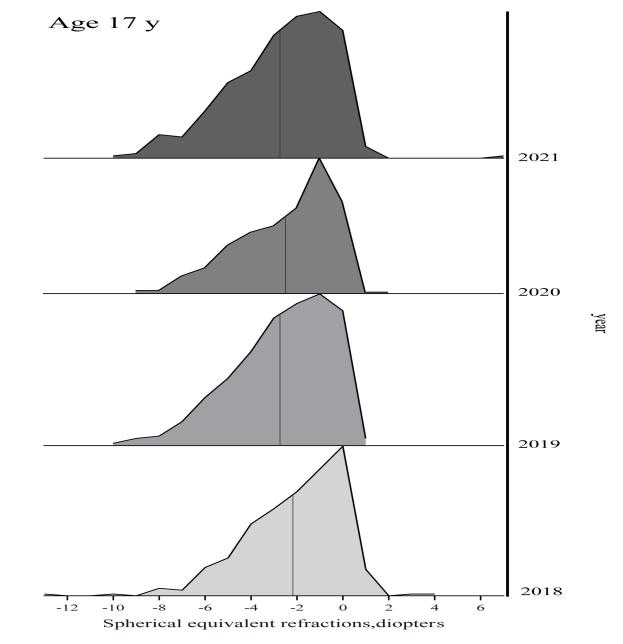

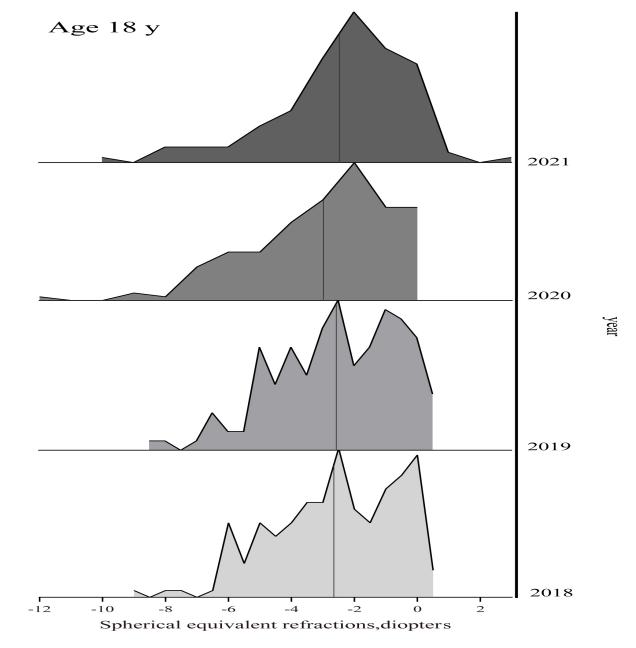


**FIGURE S3. Frequency distribution diagram of non-cycloplegic spherical equivalent refraction (SER) in the right eye at all ages at consecutive four years.**

The diagram depict the kernel density estimate of SER in the right eye for each age group within each calendar year. The x-axis represents spherical equivalent refraction in diopters (D). The y-axis represents the probability density, where the area under each curve sums to 1 for that specific age-year cohort. And the height at any point indicates the relative concentration of individuals around that specific SER value. The vertical line within each curve marks the cohort's mean SER. The visualization depicts the shifting refractive landscape: a persistent but shrinking emmetropic peak alongside a broadening left-skewed tail, illustrating the concurrent decline in the emmetropic subpopulation and the growth of the myopic majority. The progressive leftward shift of both the mean SER (vertical line) and the distribution's mass across age and calendar years visually quantifies the population-level myopic shift.

**TABLE S3. The proportion of different refractive statuses based on non-cycloplegic spherical equivalent refraction (SER) at different age groups (5-18 years old).**

| Age, y | Refractive State | 2018 | | 2019 | 2020 | 2021 |
| --- | --- | --- | --- | --- | --- | --- |
|  |  | Number (%) | | | | |
| 5 |  |  | |  |  |  |
|  | No myopia | 166 (93.26%) | | 269 (86.50%) | 220 (85.27%) | 226 (85.28%) |
|  | Mild myopia | 11 (6.18%) | | 41 (13.18%) | 38 (14.73%) | 38 (14.34%) |
|  | Moderate myopia | 1 (0.56%) | | 1 (0.32%) | NA | 1 (0.38%) |
|  | High myopia | NA | | NA | NA | NA |
| 6 |  |  | |  |  |  |
|  | No myopia | 418 (88.94%) | | 330 (83.33%) | 383 (88.05%) | 338 (84.50%) |
|  | Mild myopia | 48 (10.21%) | | 65 (16.41%) | 51 (11.72%) | 60 (15.00%) |
|  | Moderate myopia | 2 (0.43%) | | 1 (0.25%) | 1 (0.23%) | 2 (0.50%) |
|  | High myopia | 2 (0.43%) | | NA | NA | NA |
| 7 |  |  | |  |  |  |
|  | No myopia | 340 (86.29%) | | 361 (82.23%) | 337 (76.07%) | 324 (81.00%) |
|  | Mild myopia | 49 (12.44%) | | 74 (16.86%) | 104 (23.48%) | 73 (18.25%) |
|  | Moderate myopia | 3 (0.76%) | | 2 (0.46%) | 1 (0.23%) | 1 (0.25%) |
|  | High myopia | 2 (0.51%) | | 2 (0.46%) | 1 (0.23%) | 2 (0.50%) |
| 8 |  |  | |  |  |  |
|  | No myopia | 319 (78.77%) | | 328 (75.06%) | 325 (70.96%) | 265 (64.63%) |
|  | Mild myopia | 82 (20.25%) | | 96 (21.97%) | 123 (26.86%) | 136 (33.17%) |
|  | Moderate myopia | 4 (0.99%) | | 11 (2.52%) | 7 (1.53%) | 7 (1.71%) |
|  | High myopia | NA | | 2 (0.46%) | 3 (0.66%) | 1 (0.49%) |
| 9 |  |  | |  |  |  |
|  | No myopia | 290 (69.71%) | | 263 (64.46%) | 262 (59.14%) | 251 (56.03%) |
|  | Mild myopia | 113 (27.16%) | | 127 (31.13%) | 156 (35.21%) | 176 (39.29%) |
|  | Moderate myopia | 11 (2.64%) | | 16 (3.92%) | 22 (4.97%) | 18 (4.02%) |
|  | High myopia | 2 (0.48%) | | 2 (0.49%) | 3 (0.68%) | 3 (0.67%) |
| 10 |  |  | |  |  |  |
|  | No myopia | 252 (58.33%) | | 228 (50.89%) | 179 (43.77%) | 174 (41.73%) |
|  | Mild myopia | 143 (33.10%) | | 180 (40.18%) | 190 (46.45%) | 199 (47.72%) |
|  | Moderate myopia | 33 (7.64%) | | 35 (7.81%) | 38 (9.29%) | 42 (10.70%) |
|  | High myopia | 4 (0.93%) | | 5 (1.12%) | 2 (0.49%) | 2 (0.48%) |
| 11 |  |  | |  |  |  |
|  | No myopia | 216 (48.00%) | | 165 (36.67%) | 161 (35.46%) | 113 (28.39%) |
|  | Mild myopia | 188 (41.78%) | | 228 (50.67%) | 222 (48.90%) | 211 (53.02%) |
|  | Moderate myopia | 43 (9.56%) | | 49 (10.89%) | 60 (13.22%) | 68 (17.09%) |
|  | High myopia | 3 (0.67%) | | 8 (1.78%) | 11 (2.42%) | 6 (1.51%) |
| 12 |  |  |  | |  |  |
|  | No myopia | 123 (33.24%) | 128 (31.60%) | | 106 (27.04%) | 102 (24.64%) |
|  | Mild myopia | 179 (48.38%) | 196 (48.40%) | | 185 (47.19%) | 220 (53.14%) |
|  | Moderate myopia | 60 (16.22%) | 68 (16.79%) | | 91 (23.21%) | 78 (18.84%) |
|  | High myopia | 8 (2.16%) | 13 (3.21%) | | 10 (2.55%) | 14 (3.38%) |
| 13 |  |  |  | |  |  |
|  | No myopia | 122 (32.88%) | 100 (26.11%) | | 93 (22.14%) | 64 (16.71%) |
|  | Mild myopia | 192 (51.75%) | 188 (49.09%) | | 213 (50.71%) | 215 (56.14%) |
|  | Moderate myopia | 51 (13.75%) | 84 (21.93%) | | 102 (24.29%) | 84 (21.93%) |
|  | High myopia | 6 (1.62%) | 11 (2.87%) | | 12 (2.86%) | 20 (5.22%) |
| 14 |  |  |  | |  |  |
|  | No myopia | 83 (21.50%) | 64 (19.22%) | | 62 (17.22%) | 76 (19.59%) |
|  | Mild myopia | 199 (51.55%) | 178 (53.45%) | | 181 (50.28%) | 216 (55.67%) |
|  | Moderate myopia | 87 (22.54%) | 70 (21.02%) | | 100 (27.78%) | 76 (19.59%) |
|  | High myopia | 17 (4.40%) | | 21 (6.31%) | 17 (4.72%) | 20 (5.15%) |
| 15 |  |  | |  |  |  |
|  | No myopia | 62 (19.20%) | | 53 (14.13%) | 56 (14.89%) | 41 (11.68%) |
|  | Mild myopia | 153 (47.37%) | | 167 (44.53%) | 186 (49.47%) | 183 (52.14%) |
|  | Moderate myopia | 91 (28.17%) | | 125 (33.33%) | 111 (29.52%) | 105 (29.91%) |
|  | High myopia | 17 (5.26%) | | 30 (8.00%) | 23 (6.12%) | 22 (6.27%) |
| 16 |  |  | |  |  |  |
|  | No myopia | 58 (16.96%) | | 39 (11.11%) | 45 (11.34%) | 46 (12.43%) |
|  | Mild myopia | 158 (46.20%) | | 147 (41.88%) | 199 (50.13%) | 173 (46.76%) |
|  | Moderate myopia | 113 (33.04%) | | 127 (36.18%) | 118 (29.72%) | 117 (31.62%) |
|  | High myopia | 13 (3.80%) | | 38 (10.83%) | 35 (8.82%) | 34 (9.19%) |
| 17 |  |  | |  |  |  |
|  | No myopia | 60 (17.39%) | | 37 (10.91%) | 36 (10.68%) | 36 (10.43%) |
|  | Mild myopia | 162 (46.96%) | | 152 (44.84%) | 166 (49.26%) | 157 (45.51%) |
|  | Moderate myopia | 104 (30.14%) | | 118 (34.81%) | 109 (32.34%) | 117 (33.91%) |
|  | High myopia | 19 (5.51%) | | 32 (9.44%) | 26 (7.72%) | 35 (10.14%) |
| 18 |  |  | |  |  |  |
|  | No myopia | 23 (12.50%) | | 17 (11.81%) | 15 (8.62%) | 13 (10.83%) |
|  | Mild myopia | 80 (43.48%) | | 70 (48.61%) | 82 (47.13%) | 65 (54.17%) |
|  | Moderate myopia | 68 (36.96%) | | 48 (33.33%) | 53 (30.46%) | 33 (27.50%) |
|  | High myopia | 13 (7.07%) | | 9 (6.25%) | 24 (13.79%) | 9 (7.50%) |

Severity grades (based on non-cycloplegic SER at least one eye): Non-myopia > -0.50 D; Mild myopia: -3.00 D < SER ≤ -0.50 D; Moderate myopia: -6.00 D < SER ≤ -3.00 D; High myopia: SER ≤ -6.00 D.
